# Viable mutations of mouse midnolin suppress B cell malignancies

**DOI:** 10.1084/jem.20232132

**Published:** 2024-04-16

**Authors:** Xue Zhong, Nagesh Peddada, James J. Moresco, Jianhui Wang, Yiao Jiang, Jonathan J. Rios, Eva Marie Y. Moresco, Jin Huk Choi, Bruce Beutler

**Affiliations:** 1 Center for the Genetics of Host Defense, University of Texas Southwestern Medical Center, Dallas, TX, USA; 2 Center for Pediatric Bone Biology and Translational Research, Scottish Rite for Children, Dallas, TX, USA; 3 McDermott Center for Human Growth and Development, University of Texas Southwestern Medical Center, Dallas, TX, USA; 4 Department of Pediatrics, University of Texas Southwestern Medical Center, Dallas, TX, USA; 5 Department of Orthopaedic Surgery, University of Texas Southwestern Medical Center, Dallas, TX, USA

## Abstract

In a genetic screen, we identified two viable missense alleles of the essential gene *Midnolin* (*Midn*) that were associated with reductions in peripheral B cells. Causation was confirmed in mice with targeted deletion of four of six MIDN protein isoforms. MIDN was expressed predominantly in lymphocytes where it augmented proteasome activity. We showed that purified MIDN directly stimulated 26S proteasome activity in vitro in a manner dependent on the ubiquitin-like domain and a C-terminal region. MIDN-deficient B cells displayed aberrant activation of the IRE-1/XBP-1 pathway of the unfolded protein response. Partial or complete MIDN deficiency strongly suppressed Eμ-Myc–driven B cell leukemia and the antiapoptotic effects of Eμ-BCL2 on B cells in vivo and induced death of Sp2/0 hybridoma cells in vitro, but only partially impaired normal lymphocyte development. Thus, MIDN is required for proteasome activity in support of normal lymphopoiesis and is essential for malignant B cell proliferation over a broad range of differentiation states.

## Teaser

*Midnolin* is an essential gene with previously unknown effects in vivo. This paper shows that midnolin stimulates proteasome activity necessary for lymphopoiesis and B cell cancer growth in mice.

## Introduction

In 2000, midnolin (MIDN) was named for its site of expression, visualized in the midbrain of day 12.5 mouse embryos with a gene-trapped lacZ insertion in the *Midn* locus, and its localization to the nucleolus in transfected CHO cells (Tsukahara et al., 2000). More than a decade later, yeast two-hybrid screening showed an interaction between MIDN and glucokinase, a pancreatic beta cell glucose sensor, which was confirmed for full-length MIDN, and its ubiquitin-like (UBL) domain in mammalian two-hybrid analyses (Hofmeister-Brix et al., 2013). However, no further work on MIDN in the pancreas has been reported. Rather, several reports have focused on a putative association between *MIDN* gene copy number reductions and sporadic Parkinson’s disease (Billingsley et al., 2020; Obara et al., 2017, 2019, 2020; Obara and Ishii, 2018; Sagehashi et al., 2022; Sato et al., 2023), which has been disputed (Billingsley et al., 2020). Knockdown of MIDN in rat PC12 cells, a cell line derived from the adrenal gland, resulted in downregulated expression of the E3 ubiquitin ligase Parkin and the transcription factor ATF4, but the neurological significance of this is not known (Obara et al., 2017). Using CRISPR targeting in HEK293 cells in vitro, MIDN was recently implicated in ubiquitin-independent recruitment of proteins to the proteasome for degradation, particularly targeting transcription factors encoded by immediate-early genes (Gu et al., 2023). However, the physiological function of the MIDN–proteasome pathway at the cell, tissue, and organism levels has remained unknown, likely due to the lack of a viable *Midn* null mouse model (Groza et al., 2023).

We discovered viable *N*-ethyl-*N*-nitrosourea (ENU)–induced missense alleles of *Midn* in a forward genetic screen for altered functions or frequencies of lymphocytes in the blood of mice. Here, we report the immune phenotypes of these mice and mice with viable targeted deletions of multiple MIDN protein isoforms. We show that MIDN is expressed in lymphoid tissues, particularly in lymphocytes, and that MIDN interacts with and is required for proteasome activity in lymphocytes. We also show that mice homozygous for a viable hypomorphic allele of MIDN are impervious to an otherwise lethal B cell malignancy.

## Results

### Altered B cell and T cell frequencies in the blood and impaired antibody responses caused by mutations in *Midn*

We conducted a genetic screen of adult third-generation (G3) C57BL/6J mice carrying heterozygous and homozygous mutations induced by ENU in male G0 mice (Wang et al., 2015, 2018; Xu et al., 2021). To identify genes necessary for immune cell development, maintenance, or function, we performed flow cytometric analyses of immune cell frequencies in the peripheral blood and measured antibody responses to immunization with a T cell–dependent (TD) antigen (alum-precipitated ovalbumin) or a T cell–independent (TI) antigen (NP-Ficoll) (Wang et al., 2015; Xu et al., 2021). Aberrant phenotypes (e.g., altered lymphocyte frequencies) were ascribed to particular mutations by automated meiotic mapping (Wang et al., 2015; Xu et al., 2021).

**Figure 1.**
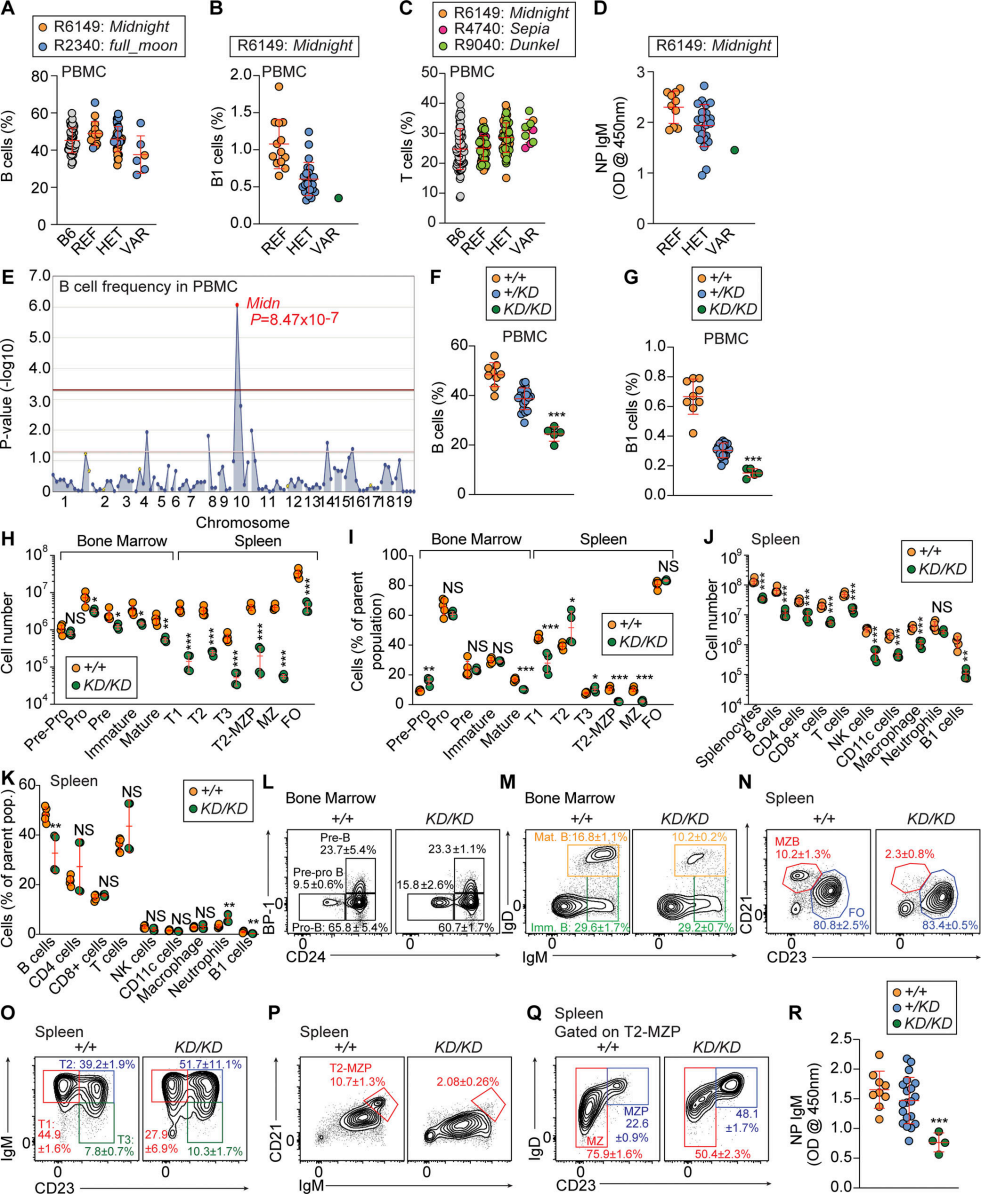
**Reduced B cells, B-1 B cells, and antibody responses caused by mutations in *Midn*. (A)** The frequency of peripheral blood B cells in G3 descendants of two independent G1 male founders (G1), with REF (*+/+*), HET (mutant/+), or VAR (mutant/mutant) genotypes for *Midn* (*n* = 48 B6, 1 *Midnight/Midnight*, 23 *Midnight/+*, 13 WT littermates and 5 *fullmoon/fullmoon*, 14 *fullmoon/+*, 2 WT littermates). **(B)** The frequency of peripheral blood B-1 B cells in G3 mice of the *Midnight* pedigree carrying the WT or *Midnight* (*Mid*) allele of *Midn* (*n* = 1 *Midnight/Midnight* [VAR], 23 *Midnight/+* [HET], 13 WT [REF] littermates). **(C)** The frequency of peripheral blood T cells in G3 mice of the *Midnight*, *Sepia*, or *Dunkel* pedigrees, with REF (*+/+*), HET (mutant/+), or VAR (mutant/mutant) genotypes for *Midn.* (*n* = 68 B6; *Midnight*: 13 REF, 23 HET, 1 VAR; *Sepia*: 9 REF, 6 HET, 3 VAR; *Dunkel*: 26 REF, 23 HET, 5 VAR littermates). **(D)** TI antibody responses of G3 mice of the *Midnight* pedigree carrying the WT or *Midnight* (*Mid*) allele of *Midn* (*n* = 1 *Midnight/Midnight* [VAR], 23 *Midnight/+* [HET], 13 WT [REF] littermates). Data are represented as absorbance at 450 nm. **(E)** Manhattan plot showing P values (−log_10_, y axis) plotted versus the chromosomal positions of mutations (x axis) identified in the G1 founders of the affected pedigrees. The *Midnight* and *full_moon* pedigrees were combined for linkage analysis. **(F and G)** Frequencies of B cells (F) and B-1 B cells (G) in peripheral blood of 15-wk-old *Midn*^*KD/KD*^, *Midn*^*+/KD*^, and WT littermates (*n* = 5 *KD/KD*, 20 *+/KD*, 9 WT littermates). **(H and I)** Numbers (H) and frequencies (I) of B cell subpopulations in the bone marrow and spleen of 8-wk-old *Midn*^*KD/KD*^ and WT littermates (*n* = 4 *KD/KD*, 5 WT littermates). **(J and K)** Numbers (J) and frequencies (K) of splenocytes and the indicated immune cell populations in the spleen of 8-wk-old *Midn*^*KD/KD*^ and WT littermates (*n* = 4 *KD/KD*, 5 WT littermates). **(L–Q)** Representative flow cytometry plots showing B cell development in the bone marrow (L and M) and spleen (N–Q) of 8-wk-old *Midn*^*KD/KD*^ and WT littermates (*n* = 4 *KD/KD*, 5 WT littermates). **(R)** T cell–independent antibody responses of 15-wk-old *Midn*^*KD/KD*^, *Midn*^*+/KD*^, and WT littermates after immunization with NP-Ficoll (*n* = 4 *KD/KD*, 20 *+/KD*, 9 WT littermates). Data are represented as absorbance at 450 nm. Flow cytometry gating strategies to analyze B cell development in the bone marrow and spleen are shown in Fig. S3 of Choi et al. (2020). Flow cytometry gating strategies to detect B cells, T cells, CD4 T cells, CD8 T cells, NK cells, CD11c^+^ cells, macrophages, neutrophils, and B-1 B cells in blood or spleen are shown in Fig. S17 A of Zhong et al. (2023). Data are representative of one experiment (A–D, F, G, and R) or two independent experiments (H–Q). Data points represent individual mice (A–D, F–K, and R). Error bars indicate SD. P values were determined by one-way analysis of variance (ANOVA) with Dunnett’s multiple comparisons (F, G, and R) or Student’s *t* test (H–K). * P < 0.05; ** P < 0.01; *** P < 0.001; NS, not significant.

**Figure S1.**
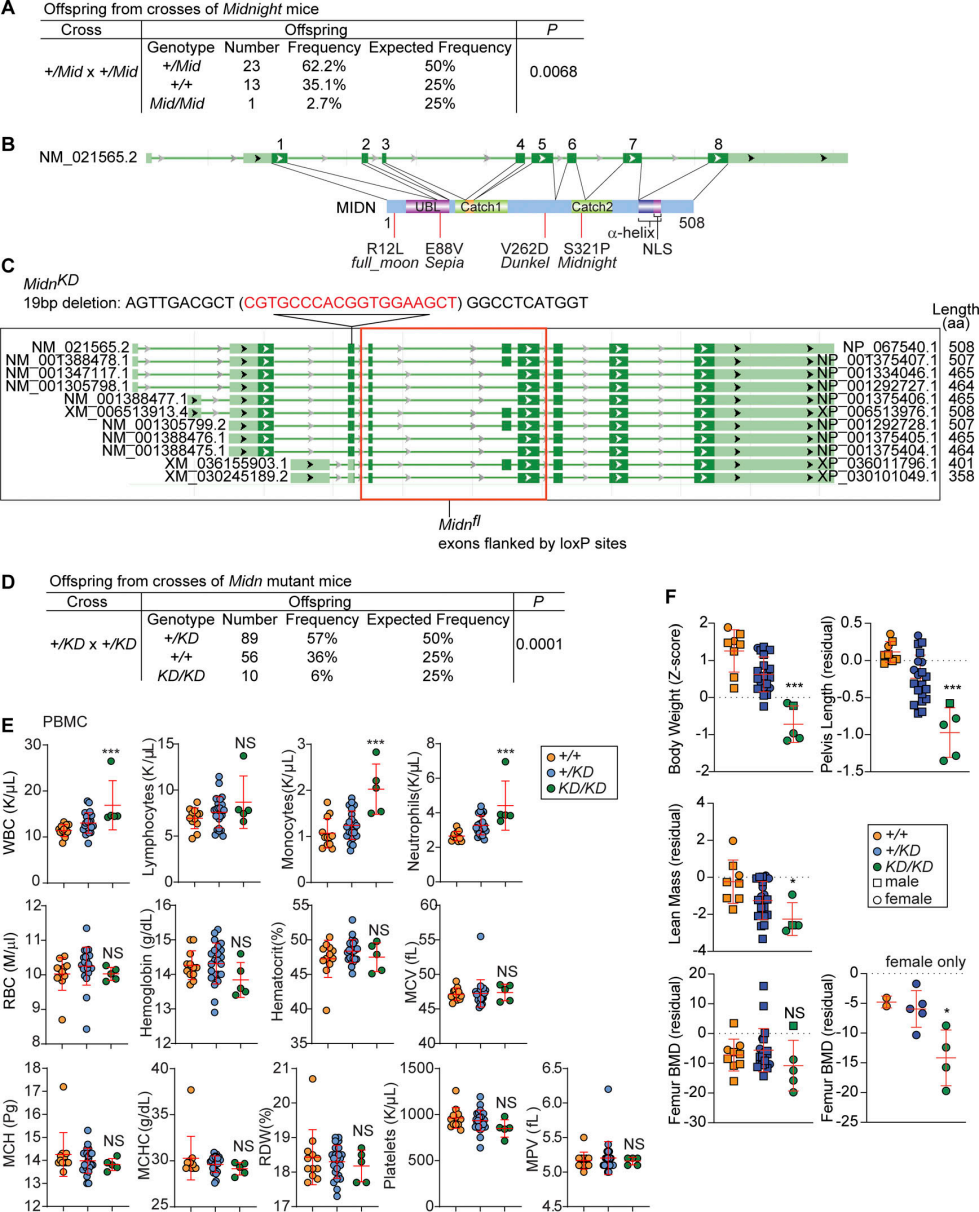
**Offspring from *Midn***^***+/Mid***^ **or *Midn***^***+/KD***^ **crosses, design of *Midn***^***fl***^ **and *Midn***^***KD***^ **alleles, and complete blood counts, body weight, and bone phenotypes in *Midn***^***KD/KD***^ **mice. (A)** Number and frequency of offspring from *Midn*^*+/Mid*^ × *Midn*^*+/Mid*^ crosses at weaning (28 days of age). A chi-square test with the appropriate degrees of freedom was used to calculate P value. **(B)** Mouse MIDN, longest isoform (508 aa, NP_067540.1), and its coding exons (NM_021565.2). Missense mutations identified in screening are indicated below. Catch1 and Catch2, regions forming the substrate-binding Catch domain identified in Gu et al. (2023); α-helix, proteasome interaction domain identified in Gu et al. (2023); NLS, nuclear localization signal. The orange region within Catch1 is missing in the 465-aa isoform. **(C)** *Midn* transcript isoforms (NCBI Gene Database). The position of the 19-bp deletion in the *Midn*^*KD*^ allele is shown above and the exons flanked by loxP sites in the *Midn*^*fl*^ allele are shown below. Length of each WT protein product is indicated on the right. **(D)** Number and frequency of offspring from *Midn*^*+/KD*^ × *Midn*^*+/KD*^ crosses at weaning (28 days of age). A chi-square test with the appropriate degrees of freedom was used to calculate P value. **(E)** Complete blood counts in 10-wk-old *Midn*^*KD/KD*^, *Midn*^*+/KD*^, and WT littermates (*n* = 5 *KD/KD*, 23 *+/KD*, 12 WT littermates). **(F)** Body weight Z-score, and residuals for lean mass, pelvis length, and femur bone mineral density (BMD) of 10-wk-old *Midn*^*KD/KD*^, *Midn*^*+/KD*^, and WT littermates (*n* = 5 *KD/KD* [4 females and 1 male], 20 *+/KD* [5 females and 15 males], 8 WT littermates [2 females and 6 males]). Residual differences (residual) between values measured in the indicated mice and expected values for a large age- and sex-matched population (n ∼ 25,300 C57BL/6J and G3 mice) are plotted for lean mass, pelvis length, and femur BMD (Rios et al., 2021); a residual equal to zero indicates no variation from expected. Data are representative of one (F) or two (E) independent experiments. Data points represent individual mice (E and F). Error bars indicate SD. P values were determined by one-way analysis of variance (ANOVA) with Dunnett’s multiple comparisons (E and F). * P < 0.05; *** P < 0.001; NS, not significant.

We identified a missense mutation in the gene *Midn* that was associated with reduced B cell and B-1 B cell frequencies and elevated T cell frequencies in the blood of mice from pedigree R6149 (Fig. 1, A–C). In addition, it was associated with a reduced antibody response to NP-Ficoll immunization (Fig. 1 D). The mutation, named *Midnight*, showed an additive (semidominant) effect on these phenotypes. Homozygous *Midnight* mice were born at lower than expected frequencies (Fig. S1 A). A second missense allele of *Midn* in an unrelated pedigree (R2340) was named *full_moon* and also associated with a reduction in peripheral B cell frequency (Fig. 1 A). Both mutations were independently associated with the B cell phenotype, and combining the two pedigrees showed an even stronger mutation–phenotype association without cosegregating mutations (Fig. 1 E; P = 8.471 × 10^−7^). Two additional *Midn* mutations from two unrelated pedigrees (R4740: *Sepia* and R9040: *Dunkel*) were associated with elevated T cell frequencies in the blood, similar to the *Midnight* allele (Fig. 1 C). Among the four *Midn* missense mutations we identified (Fig. S1 B), only *Midnight* was associated with the four phenotypes (Fig. 1, A–D); *full_moon* was associated with only the B cell phenotype (Fig. 1 A), whereas *Sepia* and *Dunkel* were associated with only the T cell phenotype (Fig. 1 C).

Since complete deficiency for *Midn* is lethal prior to weaning age (Groza et al., 2023), we generated a CRISPR/Cas9-based “knockdown” strain of *Midn* (*Midn*^*KD*^ or *KD*) in which 9 of 11 *Midn* transcript isoforms corresponding to four of six protein isoforms of *Midn* were deleted on a clean C57BL/6J background (Fig. S1 C). Two isoforms (401-aa and 358-aa) lacking the UBL domain were intact in *Midn*^*KD/KD*^ mice, which were born at less than the expected Mendelian ratio (Fig. S1 D). The primary cause of death in *Midn* null or *Midn*^*KD/KD*^ mice is unknown. We observed deficiencies in the proportions of circulating B cells and B-1 B cells in *Midn*^*+/KD*^ and *Midn*^*KD/KD*^ mice, validating that the *Midn* mutations were responsible for the original screened phenotypes (Fig. 1, F and G). *Midn*^*KD/KD*^ mice had elevated blood counts of white blood cells, monocytes, and neutrophils but otherwise normal blood count parameters (Fig. S1 E). *Midn*^*KD/KD*^ mice, especially females, also displayed significantly decreased body weight, bone mineral density, and lean (muscle) mass (Fig. S1 F).

### B cell and T cell developmental defects in *Midn*^*KD/KD*^ mice

In the bone marrow, *Midn*^*KD/KD*^ mice displayed reduced numbers of B cell progenitors beginning at the pro-B stage (Fig. 1 H). Numbers of immature and mature recirculating B cells were also diminished (Fig. 1 H), but only the mature B cell population was reduced in percentage in *Midn*^*KD/KD*^ bone marrow (Fig. 1, I and L–M). Pre-pro B cell frequencies were elevated (Fig. 1 I), suggesting a possible block in maturation at this stage. *Midn*^*KD/KD*^ mouse spleens were small and both lymphoid and myeloid cell numbers were reduced, but among these populations, only B cell and B-1 B cell frequencies were diminished (Fig. 1, J and K). Follicular B cell numbers were reduced in *Midn*^*KD/KD*^ spleens (Fig. 1 H); however, the frequencies of follicular B cells were normal (Fig. 1, I and N). Transitional B cell numbers were reduced in *Midn*^*KD/KD*^ spleens (Fig. 1 H), but only the T1 subset was reduced in frequency (Fig. 1, I and O), suggesting that development from the immature stage was impaired. Marginal zone (MZ) B cells were greatly reduced in number and frequency (Fig. 1, H, I, and N) and their development to maturity was impaired (Fig. 1, I and O–Q). B-1 B cell numbers and frequencies were also reduced in *Midn*^*KD/KD*^ spleens (Fig. 1, J and K). These findings are consistent with the impaired TI antibody response to NP-Ficoll immunization in *Midn*^*KD/KD*^ mice (Fig. 1 R).

**Figure 2.**
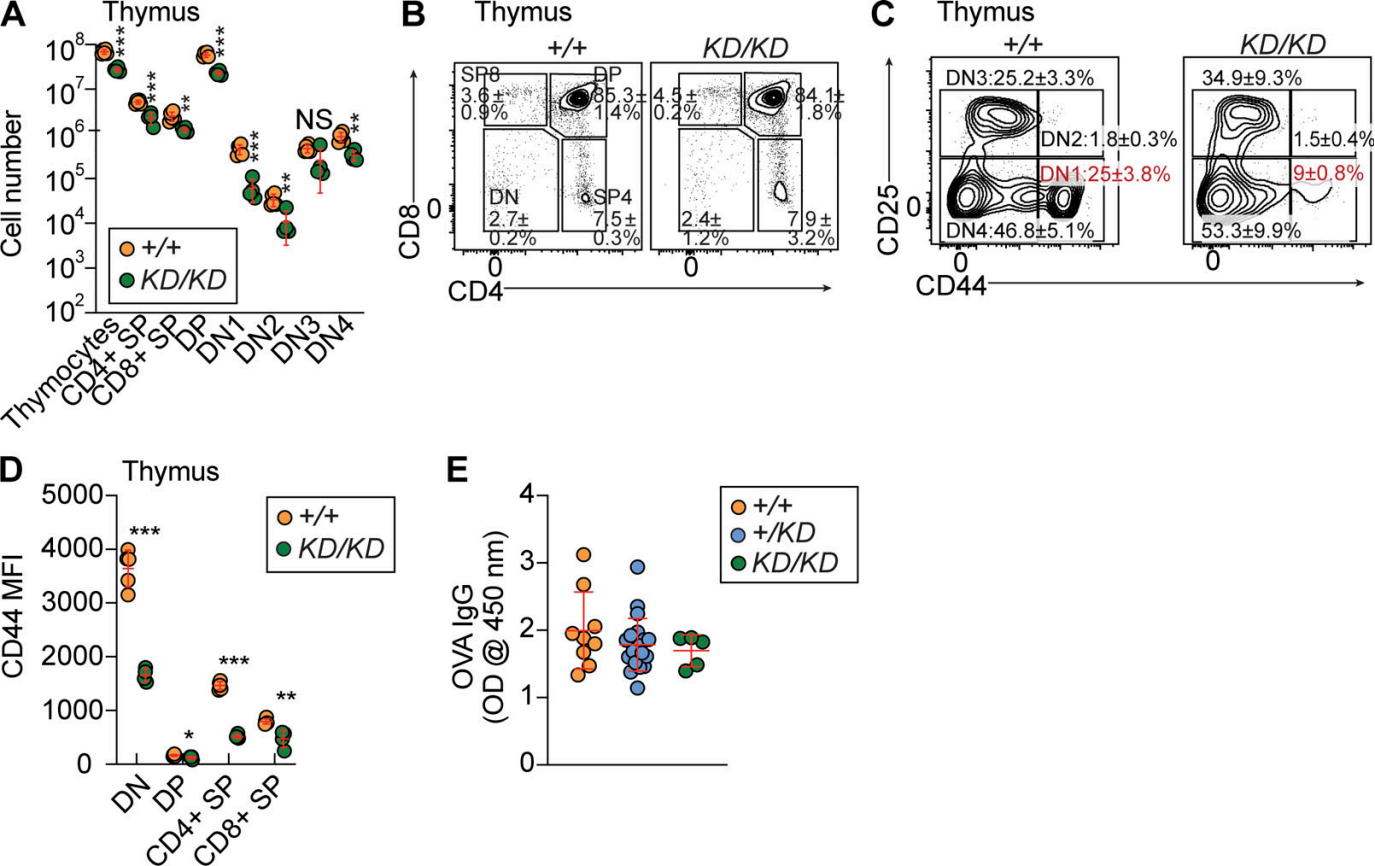
**Reduced thymic T cells in *Midn***^***KD/KD***^ **mice. (A)** Numbers of thymocyte subpopulations in the thymus of 10-wk-old *Midn*^*KD/KD*^ and WT littermates (*n* = 4 *KD/KD*, 5 WT littermates). **(B and C)** Representative flow cytometry plots showing T cell developmental stages in the thymus of 10-wk-old *Midn*^*KD/KD*^ and WT littermates (*n* = 4 *KD/KD*, 5 WT littermates). Frequencies of DN, DP, and SP thymocytes in *Midn*^*KD/KD*^ mice were normal. The percentage of DN1 thymocytes was reduced in *Midn*^*KD/KD*^ mice compared with WT mice. **(D)** Flow cytometry analysis of surface CD44 expression (mean fluorescence intensity, MFI) on thymocytes of 10-wk-old *Midn*^*KD/KD*^ and WT littermates (*n* = 4 *KD/KD*, 5 WT littermates). **(E)** TD antibody responses of 10-wk-old *Midn*^*KD/KD*^, *Midn*^*+/KD*^, and WT littermates after immunization with aluminum hydroxide-precipitated ovalbumin (OVA) (*n* = 5 *KD/KD*, 20 *+/KD*, 9 WT littermates). Data are represented as absorbance at 450 nm. Flow cytometry gating strategies to analyze T cell development in the thymus are shown in Fig. S2 of Choi et al. (2020). Data are representative of two independent experiments. Data points represent individual mice (A, D, and E). Error bars indicate SD. P values were determined by one-way analysis of variance (ANOVA) with Dunnett’s multiple comparisons (E) or Student’s *t* test (A and D). * P < 0.05; ** P < 0.01; *** P < 0.001; NS, not significant. DN1-4, stage 1–4 DN.

*Midn*^*KD/KD*^ mice displayed smaller thymi and diminished numbers of developing and mature thymocytes compared with WT littermates (Fig. 2 A). However, once they passed the DN1 stage, thymocyte development was largely normal in *Midn*^*KD/KD*^ mice despite reduced CD44 expression on double-positive (DP), double-negative (DN), and single-positive (SP) thymocytes (Fig. 2, B–D). TD antibody responses were normal in *Midn*^*KD/KD*^ mice (Fig. 2 E).

**Figure 3.**
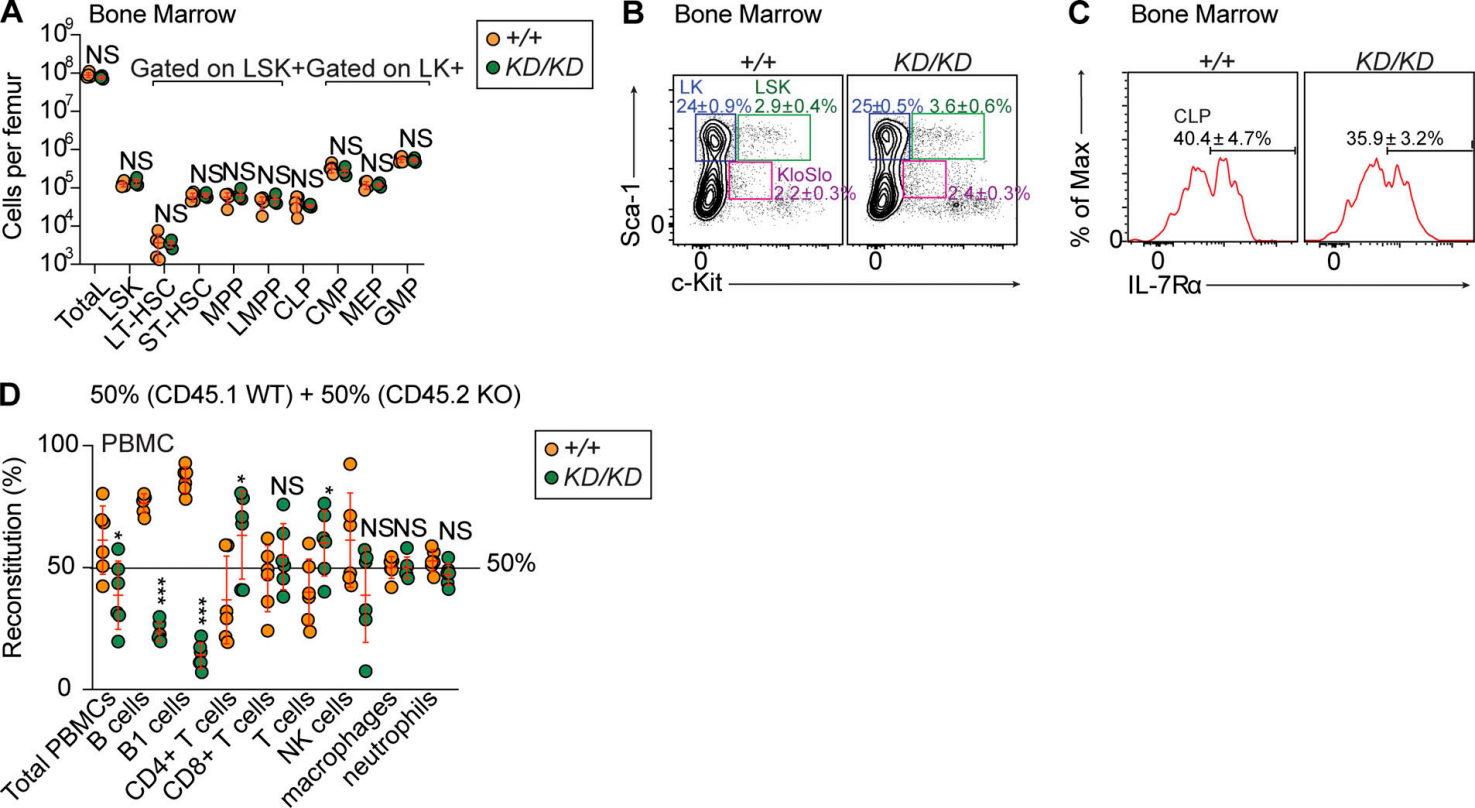
**Normal HSPC populations in *Midn***^***KD/KD***^ **mice. (A–C)** Numbers (A) and representative flow cytometry plots of HSPC populations (B and C) in 10-wk-old *Midn*^*KD/KD*^ and WT littermates (*n* = 4 *KD/KD*, 5 WT littermates). **(D)** Repopulation of the indicated populations in the blood of lethally irradiated *Rag2*^*−/−*^ recipient mice 12 wk after transfer of a 1:1 mixture of WT and *Midn*^*KD/KD*^ bone marrow cells (*n* = 6 recipients per group). Flow cytometry gating strategies to analyze HSPC populations, except LMPP, are shown in Fig. S1 of Choi et al. (2020). Flow cytometry gating strategies to analyze LMPP are shown in Fig. S2 A of this paper. Data are representative of two independent experiments. Data points represent individual mice (A and D). Error bars indicate SD. P values were determined by Student’s *t* test (A and D). * P < 0.05; *** P < 0.001; NS, not significant.

**Figure S2.**
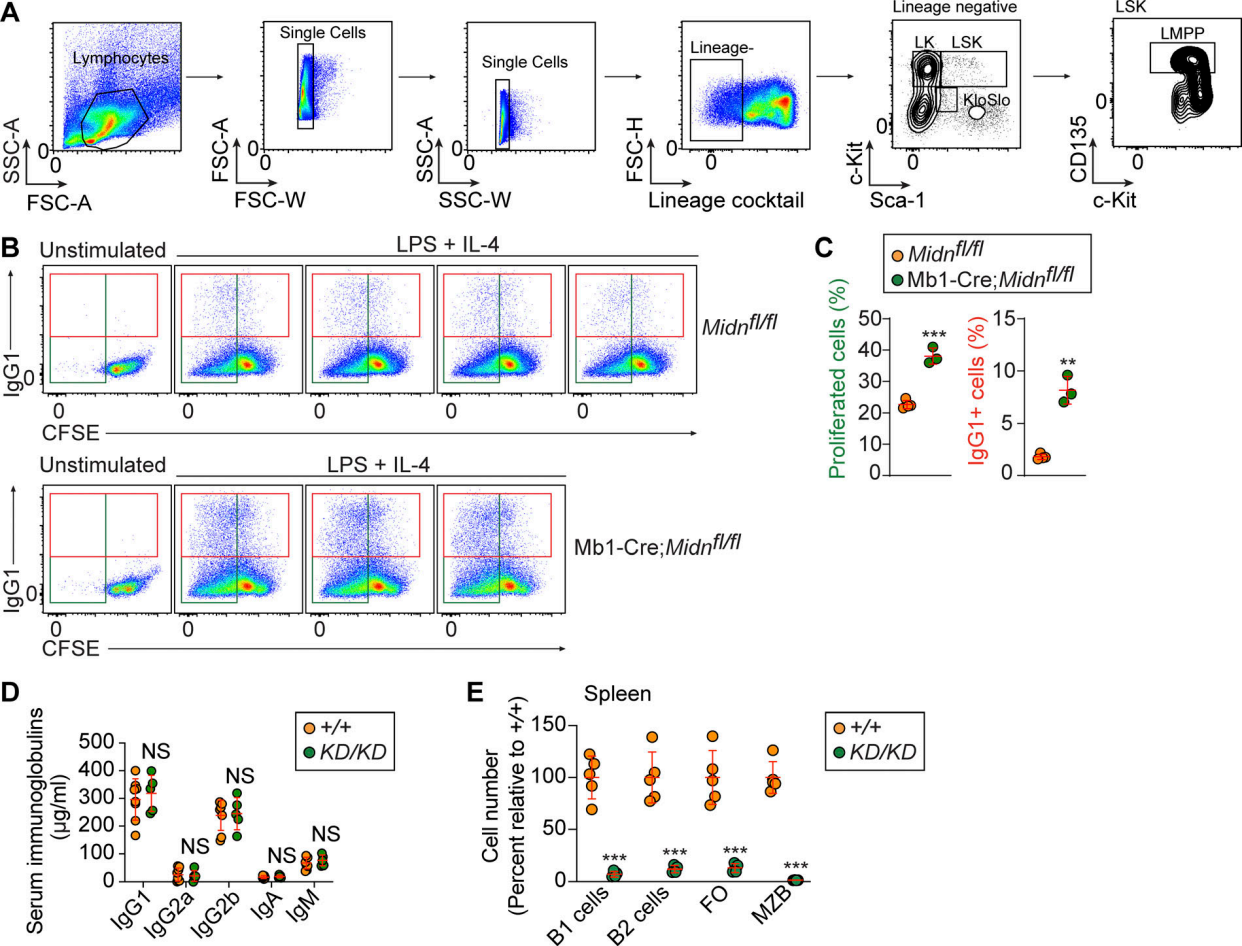
**Increased proliferation and class switching by MIDN-deficient B cells compared with WT B cells. (A)** Gating strategy for LMPP. **(B and C)** Splenic pan-B cells isolated from *Midn*^*fl/fl*^ and Mb1-Cre;*Midn*^*fl/fl*^ littermates were labeled with CFSE and then treated with LPS and IL-4 in culture (*n* = 3 Mb1-Cre;*Midn*^*fl/fl*^ and 4 *Midn*^*fl/fl*^ littermates). Representative flow cytometry plots (B) and frequencies (C) of CFSE^low^ cells (proliferated cells) and IgG1^+^ cells before (unstimulated) and after LPS and IL-4 treatment. **(D)** Concentration of immunoglobulins in the serum of 12-wk-old *Midn*^*KD/KD*^ or WT littermates (*n* = 5 *Midn*^*KD/KD*^ and 8 WT littermates). **(E)** Cell numbers of the indicated B cell populations in the spleen. Numbers in *Midn*^*KD/KD*^ spleens were normalized to numbers in WT spleens. Data are representative of two independent experiments. Data points represent individual mice (C–E). Error bars indicate SD. P values were determined by Student’s *t* test (C–E). ** P < 0.01; *** P < 0.001; NS, not significant.

Hematopoietic stem and progenitor cell (HSPC) populations were present in normal numbers and frequencies in *Midn*^*KD/KD*^ bone marrow (Fig. 3, A–C; and Fig. S2 A). Strikingly, bone marrow transplantation in which *Midn*^*KD/KD*^ (CD45.2) and WT (CD45.1) bone marrow cells were mixed 1:1 and transferred to *Rag2*^*−/−*^ mice showed reduced ability of *Midn*^*KD/KD*^ bone marrow to reconstitute B cell populations whereas other lymphoid and myeloid populations were reconstituted equally by *Midn*^*KD/KD*^ and WT bone marrow (Fig. 3 D). These data support a cell-intrinsic role for MIDN specifically in B cell development and/or maintenance.

### B cell–specific MIDN deletion shows a cell-intrinsic role in B cell development and function

**Figure 4.**
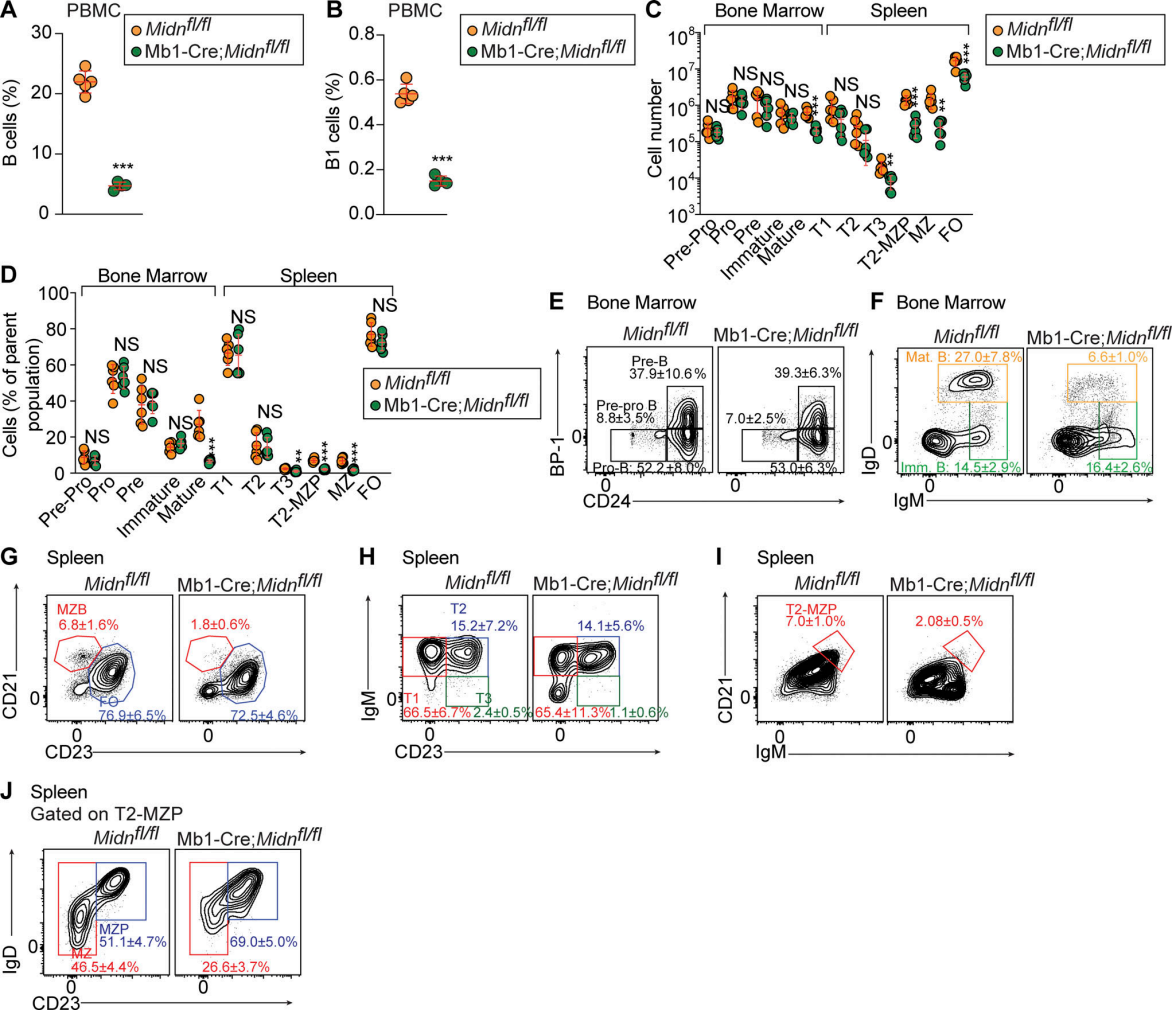
**MIDN has a cell-intrinsic role in B cell development. (A and B)** Frequencies of peripheral blood B cells (A) and B-1 B cells (B) in 8-wk-old Mb1-Cre;*Midn*^*fl/fl*^ and *Midn*^*fl/fl*^ littermates (*n* = 4 Mb1-Cre;*Midn*^*fl/fl*^, 5 *Midn*^*fl/fl*^ littermates). **(C and D)** Numbers (C) and frequencies (D) of B cell subpopulations in the bone marrow and spleen of 10-wk-old Mb1-Cre;*Midn*^*fl/fl*^ and *Midn*^*fl/fl*^ littermates (*n* = 6 Mb1-Cre;*Midn*^*fl/fl*^, 6 *Midn*^*fl/fl*^ littermates). **(E–J)** Representative flow cytometry plots showing B cell development in the bone marrow (E and F) and spleen (G–J) of 10-wk-old Mb1-Cre;*Midn*^*fl/fl*^ and *Midn*^*fl/fl*^ littermates (*n* = 6 Mb1-Cre;*Midn*^*fl/fl*^, 6 *Midn*^*fl/fl*^ littermates). Heterozygous carriers of the Mb1-Cre transgene were used. Flow cytometry gating strategies to analyze B cell development in the bone marrow and spleen are shown in Fig. S3 of Choi et al. (2020). Flow cytometry gating strategies to detect B cells and B-1 B cells in blood are shown in Fig. S17 A of Zhong et al. (2023). Data are representative of two independent experiments. Data points represent individual mice (A–D). Error bars indicate SD. P values were determined by Student’s *t* test (A–D). ** P < 0.01; *** P < 0.001; NS, not significant.

To determine the effects of MIDN deletion specifically in B cells, we generated mice in which *Midn* exons 4–6 were flanked by loxP sites (*Midn*^*fl*^; Fig. S1 C) and crossed them with transgenic mice expressing *Mb1* promoter-driven Cre recombinase. Mb1-Cre;*Midn*^*fl/fl*^ mice exhibited reduced frequencies of B cells and B-1 cells in the blood as expected (Fig. 4, A and B). In the bone marrow, mature B cells were reduced in number and frequency, but B cell progenitors and immature B cells were normal in numbers and frequencies in Mb1-Cre;*Midn*^*fl/fl*^ mice (Fig. 4, C–F). This is in contrast with *Midn*^*KD/KD*^ bone marrow, in which progenitor and immature B cell populations were reduced in numbers (Fig. 1 H). In the spleen, Mb1-Cre;*Midn*^*fl/fl*^ transitional populations showed a trend toward reduced numbers, with the T3 population statistically significantly reduced in numbers and frequencies (Fig. 4, C, D, and H). MZ precursors and MZ B cells were reduced in Mb1-Cre;*Midn*^*fl/fl*^ mice (Fig. 4, C, D, G, I, and J), similar to *Midn*^*KD/KD*^ mice. Overall, analysis of Mb1-Cre;*Midn*^*fl/fl*^ mice confirmed that MIDN has a cell-intrinsic role in splenic B cell development and suggested that MIDN may also support mature B cell homeostasis.

We also assessed the function of MIDN-deficient B cells by measuring proliferation and class switching by splenic pan-B cells treated with LPS and IL-4 in vitro. Compared with B cells from *Midn*^*fl/fl*^ mice, Mb1-Cre;*Midn*^*fl/fl*^ B cells exhibited increased proliferation and increased class switching to IgG after LPS and IL-4 treatment (Fig. S2, B and C). Together with the observation of similar concentrations of serum immunoglobulins in WT and *Midn*^*KD/KD*^ mice (Fig. S2 D), these findings suggest that B cells may be intrinsically hyperfunctional in mice with reduced or absent MIDN function. We compared the relative reductions in B-1, B-2, follicular, and MZ B cells in *Midn*^*KD/KD*^ spleens. We found that B-1 and MZ B cells were more severely reduced compared with B-2 and follicular B cells (Fig. S2 E). These findings may explain why TI but not TD antibody responses were impaired in *Midn*^*KD/KD*^ mice: The remaining hyperfunctional B-2 and follicular B cells may be sufficient to produce a TD antibody response of normal magnitude. In contrast, even with increased function, the very few MZ and B-1 B cells may not be able to produce normal levels of antibodies against a TI antigen.

### MIDN is expressed predominantly by lymphoid cells

**Figure S3.**
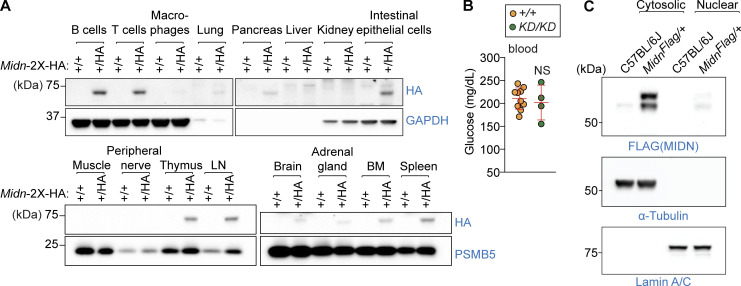
**MIDN is expressed predominantly by lymphoid cells. (A)** Immunoblot analysis of HA-tagged endogenous MIDN in lysates of the indicated tissues from 8-wk to 3-mo-old mice. GAPDH or PSMB5 were used as loading controls. **(B)** Normal blood glucose in *Midn*^*KD/KD*^ mice. Serum glucose concentration in 8-wk-old *Midn*^*KD/KD*^ and WT littermates (*n* = 4 *KD/KD*, 10 WT littermates). **(C)** Immunoblot analysis of FLAG-tagged endogenous MIDN in subcellular fractions of B cell lysates from 8-wk to 3-mo-old WT or *Midn*^*Flag/+*^ mice. α-tubulin and lamin A/C were used as cytosolic and nuclear loading controls, respectively. Data are representative of two independent experiments. Data points represent individual mice (B). Error bars indicate SD. P values were determined by Student’s *t* test (B). NS, not significant. Source data are available for this figure: SourceData FS3.

We generated mice with either a C-terminal 3x-FLAG tag or a 2x-HA tag knocked-in to the *Midn* genomic locus (*Midn*^*Flag*^ or *Midn*^*HA*^). Immunoblot analysis of tissues from adult *Midn*^*+/HA*^ mice revealed prominent MIDN expression in primary and secondary lymphoid tissues (bone marrow, thymus, spleen, and lymph nodes) and specifically in B cells and T cells (Fig. S3 A). Expression was also detected in intestinal epithelial cells, and very low expression in the lung, pancreas, brain, adrenal gland, and possibly in macrophages, whereas MIDN was absent from the liver, kidney, muscle, and peripheral nerve (Fig. S3 A). These data are consistent with the B cell and T cell phenotypes observed in *Midn*^*KD/KD*^ mice. MIDN expression in the pancreas is consistent with the reported interaction between MIDN and glucokinase (Hofmeister-Brix et al., 2013); however, blood glucose was normal in *Midn*^*KD/KD*^ mice (Fig. S3 B). By cellular fractionation of unstimulated splenic B cells from *Midn*^*Flag/+*^ mice, we demonstrated that MIDN was predominantly localized in the cytosol rather than the nucleus (Fig. S3 C).

### Proteasomes interact with and require MIDN for activity in unstimulated B cells

**Figure 5.**
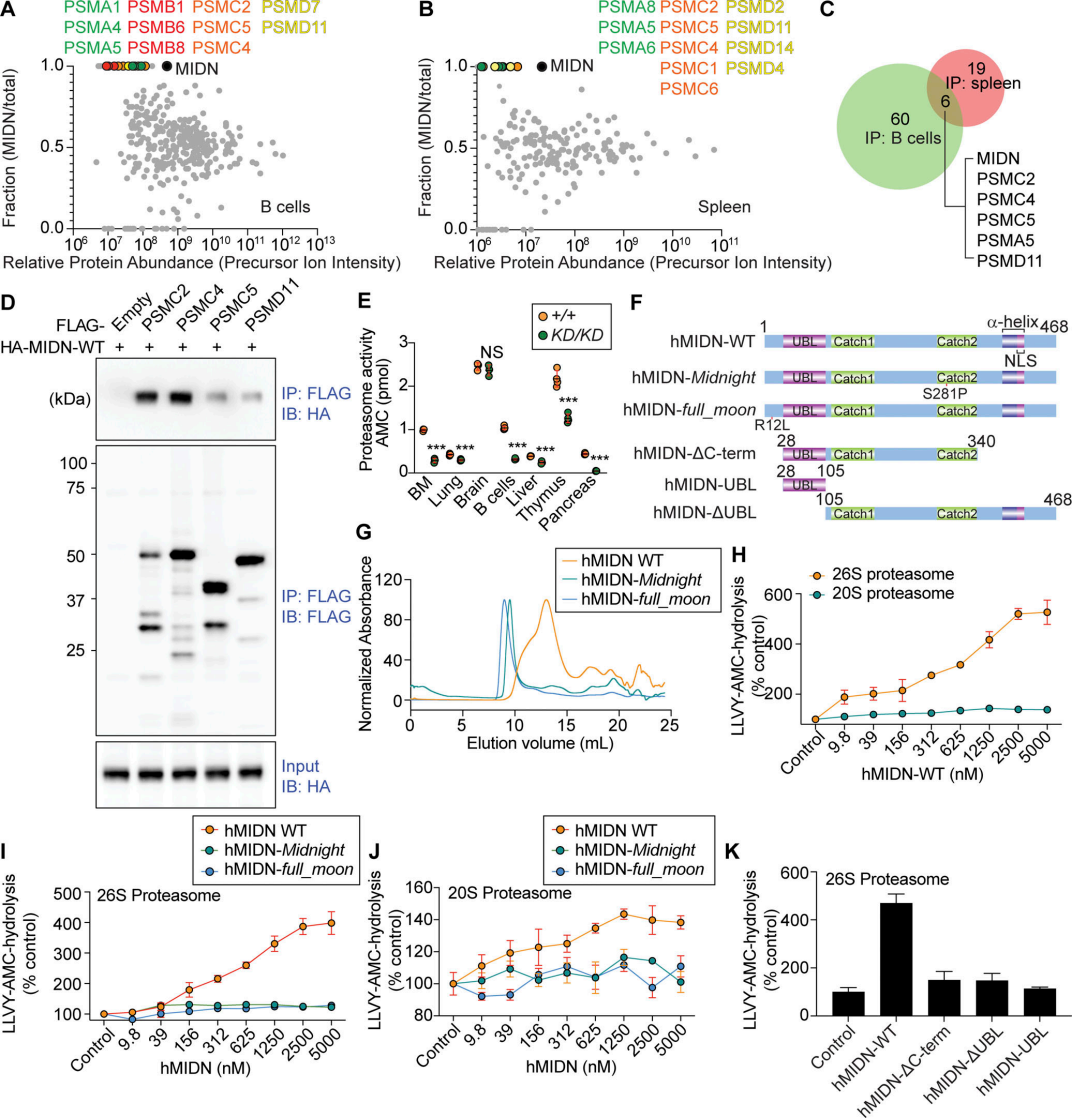
**Proteasomes interact with and require MIDN for activity in B cells and MIDN stimulates proteasome activity in vitro. (A and B)** MIDN interactors identified by co-IP combined with LC-MS/MS using lysates of B cells (A) or spleen (B) from *Midn*^*Flag/Flag*^ mice or WT mice. Relative protein abundance calculated using precursor ion intensities (abundance in MIDN IP divided by the sum of abundances in MIDN and WT control IP) is plotted on the y axis. y = 0.5 indicates equivalent abundance in the MIDN IP and WT control IP. y > 0.5 indicates enrichment in the MIDN IP with y = 1 indicating the protein was exclusively detected in the MIDN IP. Proteasome components were absent in WT control samples subjected to the same pull-down procedure but lacking the knock-in FLAG tag. Proteasome 20S particle alpha subunits (green) and beta subunits (red); proteasome 19S particle base subunits (orange) and lid subunits (yellow). **(C)** Summary of MIDN interactors identified in the two experiments in A and B. **(D)** Confirmation of interactions between MIDN and 19S components. HEK293T cells transiently expressing each protein were lysed, and then lysates were mixed and subjected to immunoprecipitation using anti-FLAG M2 agarose followed by immunoblot analysis with antibodies against HA or FLAG. **(E)** Proteasome peptidase activity as measured by AMC fluorescence after hydrolysis of LLVY-AMC in lysates of the indicated tissues from *Midn*^*KD/KD*^ and WT littermates (*n* = 3 mice/genotype in bone marrow and brain, 4 mice/genotype in lung, B cells, liver, thymus, and pancreas). **(F)** Human MIDN constructs used for protein biochemistry studies. **(G)** Size exclusion chromatography profiles of hMIDN-WT, hMIDN-*Midnight*, and hMIDN-*full_moon*. **(H–J)** In vitro peptidase activity of purified 26S or 20S proteasomes (1 nM) as measured by AMC fluorescence after hydrolysis of LLVY-AMC (10 μM) in the presence of ATP (0.2 mM). Purified hMIDN-WT, hMIDN-*Midnight*, or hMIDN-*full_moon* was added at the indicated concentrations, or no hMIDN was added (Control). **(K)** In vitro peptidase activity of 26S proteasomes assayed as in H with the indicated purified hMIDN proteins added at 5 μM, or without hMIDN (Control). In H–K, activity is plotted relative to the activity of each type of proteasome without added MIDN proteins (Control, set at 100%) (*n* = 3 reactions per condition). Data are representative of one (A and B), two (D), three (G–K), or four independent experiments (E). Data points represent individual mice (E). Error bars indicate SD. P values were determined by Student’s *t* test (E and H) or one-way analysis of variance (ANOVA) with Dunnett’s multiple comparisons (I–K). *** P < 0.001; NS, not significant. Source data are available for this figure: SourceData F5.

We analyzed the interactome of endogenous 3x-FLAG tagged MIDN in splenic B cells or total splenocytes by mass spectrometry. MIDN immunoprecipitated a complex containing multiple proteasome components from both the 19S regulatory particle and the 20S core particle (Fig. 5, A–C; and Data S1 and S2). The 19S particle activates proteasome activity by opening the restrictive gate formed by α subunits of the 20S particle (Bard et al., 2018; Groll et al., 2000; Köhler et al., 2001) and recruits, unfolds, and translocates protein substrates into the 20S core for degradation (Kors et al., 2019). Nine 19S subunits (PSMC1, PSMC2, PSMC4, PSMC5, PSMD2, PSMD6, PSMD7, PSMD11, and PSMD12) and three 20S subunits (PSMA1, PSMA5, and PSMA6) were detected between the two experiments. We validated the interaction between MIDN and several proteasome components by co-immunoprecipitation (co-IP) (Fig. 5 D). These data support a functionally important interaction between MIDN and the proteasome.

We used *N*-succinyl-Leu-Leu-Val-Tyr-7-amino-4-methylcoumarin (LLVY-AMC), a fluorogenic peptide substrate for the chymotrypsin-like activity of the proteasome β5 subunit (Kisselev and Goldberg, 2005), to measure the peptidase activity of proteasomes in splenic B cell lysates from WT and *Midn*^*KD/KD*^ mice. We found an 82% reduction in peptidase activity of proteasomes in *Midn*^*KD/KD*^ B cell lysates compared with WT B cell lysates (Fig. 5 E). Using the same assay, we found diminished proteasome activity in *Midn*^*KD/KD*^ bone marrow, thymus, lung, and pancreas lysates (Fig. 5 E), consistent with the tissue expression pattern of MIDN (Fig. S3, A and B). Although we did not detect MIDN expression in the liver, we found reduced proteasome activity in *Midn*^*KD/KD*^ liver lysates. We speculate that MIDN may be expressed in a specific liver cell type but is below the level of detection in whole liver lysates. No difference in proteasome activity was detected between *Midn*^*KD/KD*^ and WT mouse brain lysates (Fig. 5 E). These data suggest that MIDN is necessary for proteasome activity in the tissues in which it is expressed.

**Figure S4.**
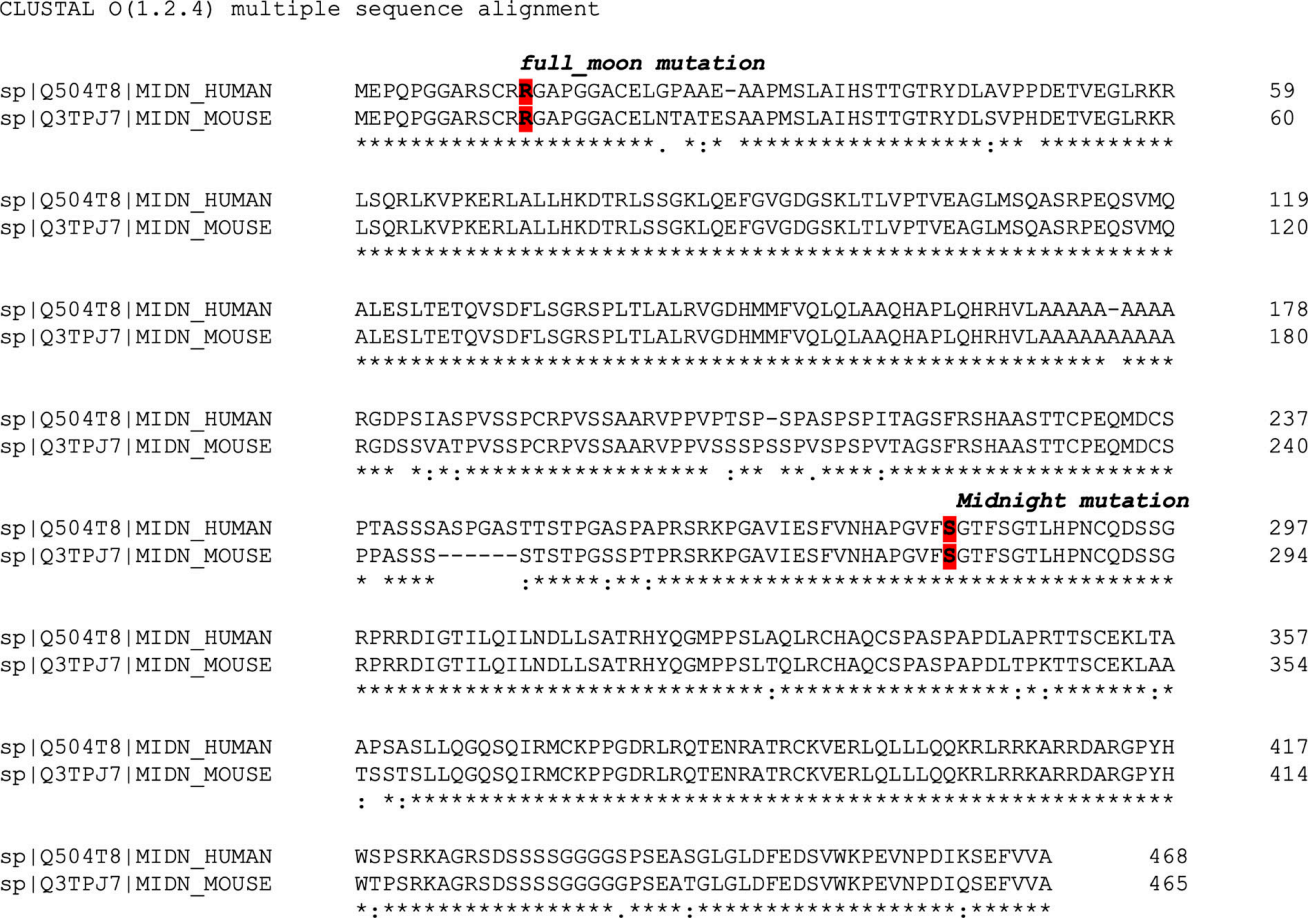
**Alignment of human and mouse MIDN.** 468-aa human MIDN (Uniprot Q504T8) and 465-aa mouse MIDN (Uniprot Q3TPJ7) were aligned using the Clustal Omega tool. The residues affected by the *Midnight* and *full_moon* mutations are indicated.

MIDN contains one region with sequence homology to a known protein domain: a UBL domain spanning aa 28–106. To test whether MIDN stimulates proteasome activity in vitro like other UBL domain–containing proteins (Kim and Goldberg, 2018), we purified full-length human (h) MIDN (468-aa isoform), point mutants, and truncated versions of hMIDN (Fig. 5 F). Human and mouse MIDN are 94% identical in amino acid sequence (Fig. S4). hMIDN with the *Midnight* or *full_moon* point mutation eluted earlier than WT hMIDN in size exclusion chromatography, suggesting the mutant proteins are less stable and tend to form aggregates in vitro (Fig. 5 G). hMIDN stimulated peptidase activity of purified human 26S proteasomes but not 20S core particles (Fig. 5 H). Mutant forms of hMIDN bearing changes orthologous to the ENU-induced changes in mouse MIDN (Fig. 5, I and J), and hMIDN-ΔC-term, hMIDN-ΔUBL, or the isolated UBL domain showed severely reduced proteasome stimulatory activity compared with full-length WT hMIDN (Fig. 5 K). These data indicate a direct stimulatory effect of hMIDN on proteasome peptidase activity in vitro that requires the presence of the 19S particle.

### MIDN mutation or deletion suppressed lymphoproliferation in models of leukemia and lymphoma

Inhibitors of the 20S core of the proteasome (bortezomib, ixazomib, and carfilzomib) were approved for the treatment of multiple myeloma or mantle cell lymphoma (Manasanch and Orlowski, 2017). The lethal effect of proteasome inhibitors on multiple myeloma is attributed in part to the induction of an apoptotic unfolded protein response (UPR) that occurs if excessive ER stress is not alleviated (Cenci et al., 2006; Obeng et al., 2006; Sha and Goldberg, 2020). An activated survival-promoting UPR, and specifically activation of the IRE-1/XBP-1 arm of the UPR, was observed in B cells from mouse models and humans with chronic lymphocytic leukemia (CLL) (Kriss et al., 2012). Based upon these findings and our data supporting the necessity of MIDN for intact proteasome activity in B cells, we tested the hypothesis that targeting MIDN in the setting of an activated UPR in B cell cancers might result in growth arrest or death of malignant B cells or plasma cells.

**Figure 6.**
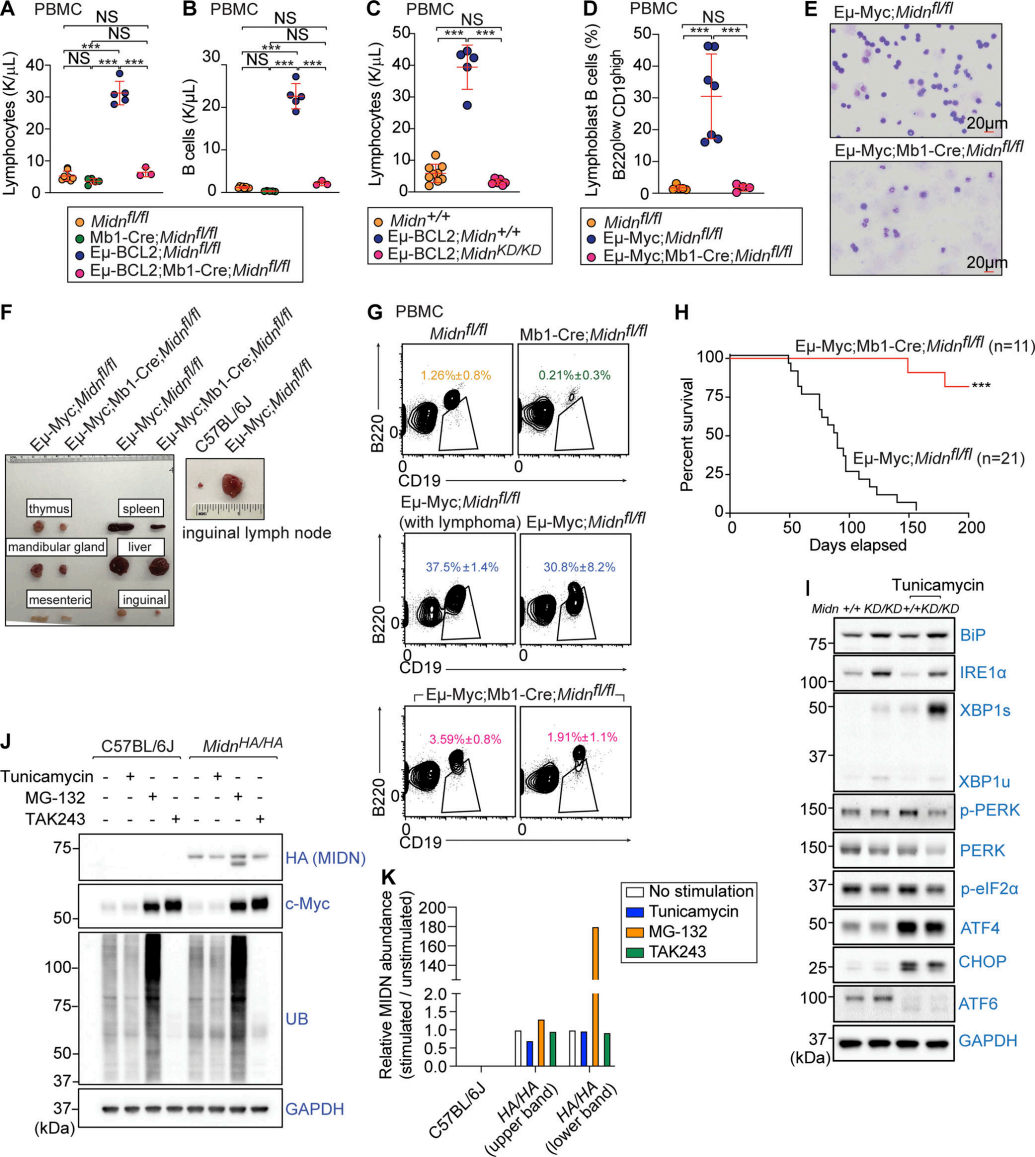
**MIDN mutation or deletion suppressed elevated B cell numbers in genetic models of leukemia and lymphoma. (A and B)** Lymphocytes (A) and B cells (B) in the blood of 8-wk-old mice with or without B cell–specific *Midn* deletion, and in Eμ-BCL2 mice with or without B cell–specific *Midn* deletion (*n* = 3 Eμ-BCL2;Mb1-Cre;*Midn*^*fl/fl*^, 5 Mb1-Cre;*Midn*^*fl/fl*^, 5 Eμ-BCL2;*Midn*^*fl/fl*^, 8 *Midn*^*fl/fl*^ littermates). **(C)** Lymphocytes in the blood of 8-wk-old Eμ-BCL2 mice on a WT or *Midn*^*KD/KD*^ background, and in WT control mice (*n* = 6 Eμ-BCL2;*Midn*^*KD/KD*^, 5 Eμ-BCL2;*Midn*^*+/+*^, 9 *Midn*^*+/+*^ littermates). **(D and E)** Frequency of B lymphoblasts (B220^low^CD19^high^) in the blood (D) and representative blood smears (E) from 10-wk-old Eμ-Myc mice with or without B cell–specific *Midn* deletion, and control *Midn*^*fl/fl*^ mice (*n* = 4 Eμ-Myc;Mb1-Cre;*Midn*^*fl/fl*^, 7 Eμ-Myc;*Midn*^*fl/fl*^, 5 *Midn*^*fl/fl*^ littermates). **(F)** Representative photograph of thymi, spleens, livers, and lymph nodes (mandibular, mesenteric, and inguinal) from 16-wk-old Eμ-Myc mice with or without B cell–specific *Midn* deletion, and control WT (C57BL/6J) mice. **(G)** Representative flow cytometry plots showing B cells in the blood of 10-wk-old Eμ-Myc mice with or without B cell–specific *Midn* deletion, and control *Midn*^*fl/fl*^ mice (*n* = 3 19-wk-old Eμ-Myc;Mb1-Cre;*Midn*^*fl/fl*^, 4 10-wk-old Eμ-Myc;Mb1-Cre;*Midn*^*fl/fl*^, 6 19-wk-old Eμ-Myc;*Midn*^*fl/fl*^ (with lymphoma), 5 10-wk-old Eμ-Myc;*Midn*^*fl/fl*^, 5 Mb1-Cre;*Midn*^*fl/fl*^, and 12 *Midn*^*fl/fl*^ littermates). Leukemic B cell precursors are CD19^+^B220^low^. Eμ-Myc;*Midn*^*fl/fl*^ mice develop lymphadenopathy at different ages (range 10–19 wk), and plots are shown both for those that already developed and have yet to develop lymphadenopathy. **(H)** Survival curve of Eμ-Myc mice with or without B cell–specific *Midn* deletion. **(I)** Immunoblot analysis of the indicated UPR proteins in lysates of WT and *Midn*^*KD/KD*^ splenic B cells after tunicamycin treatment or without treatment. Heterozygous carriers of the Mb1-Cre transgene were used in A–H. **(J and K)** Immunoblot analysis (J) and quantitation (K) of endogenous MIDN, c-Myc, ubiquitin, and GAPDH in splenic B cell lysates from WT and *Midn*^*HA/HA*^ mice after tunicamycin (3.3 μM, 6 h), MG-132 (10 μM, 6 h), or TAK-243 (500 nM, 6 h) treatment or without treatment. Data are representative of two independent experiments. Data points represent individual mice (A–D). Error bars indicate SD. P values were determined by one-way analysis of variance (ANOVA) with Dunnett’s multiple comparisons (A–D) or log-rank test (H). *** P < 0.001; NS, not significant. Source data are available for this figure: SourceData F6.

BCL2 is an inhibitor of apoptosis and accordingly, transgenic expression of human *BCL2* driven by the IgH enhancer in mice (Eμ-BCL2) induces an expansion of B cells, immunoglobulin-secreting cells, and serum immunoglobulins; all B lineage cells exhibit prolonged survival in these mice (Strasser et al., 1991). Eμ-BCL2 mice occasionally develop lymphomas (Strasser et al., 1993); moreover, in humans, a chromosomal translocation linking the *IGH* and *BCL2* loci is associated with B lymphomas and CLL (Adachi et al., 1990; Tsujimoto et al., 1985), and BCL2 is typically overexpressed in CLL (Calin et al., 2008; Kitada et al., 1998). We deleted *Midn* specifically in B cells of Eμ-BCL2;Mb1-Cre;*Midn*^*fl/fl*^ mice. No expansion of lymphocytes was observed, and B cell accumulation was greatly reduced (Fig. 6, A and B). We also observed suppression of lymphocyte counts in Eμ-BCL2 mice on a *Midn*^*KD/KD*^ background (Fig. 6 C).

Transgenic *Myc* expression driven by the IgH enhancer (Eμ-Myc) results in spontaneous lymphomas presenting as lymphadenopathy, as well as pre-B cell leukemias early in life (3–18 wk, median 9 wk of age) (Adams et al., 1985; Harris et al., 1988). We crossed Mb1-Cre;*Midn*^*fl/fl*^ mice to Eµ-Myc mice to achieve B cell–specific *Midn* deletion in this leukemia/lymphoma model. MIDN deficiency in B cells greatly reduced lymphadenopathy, lymphoblasts, and leukemic B cell precursors in the blood caused by Eμ-Myc transgene expression (Fig. 6, D–G). All Eμ-Myc transgenic mice with intact MIDN (21/21) died by 156 days of age; in contrast, all Eμ-Myc;Mb1-Cre;*Midn*^*fl/fl*^ mice (11/11) survived until at least 149 days of age, and 82% (9/11) survived past 200 days of age (Fig. 6 H). Together, these data demonstrate that either partial or complete deficiency of *Midn* suppressed the antiapoptotic effects of BCL2 overexpression and B lymphoproliferation in a Myc-driven model of B cell malignancy.

Consistent with the idea that proteasome activity downregulates the UPR and that MIDN is required for proteasome activity in B cells, we found that B cells from *Midn*^*KD/KD*^ mice displayed an elevated UPR, evidenced by increased expression of IRE1α and XBP1 (Fig. 6 I). XBP1s was further elevated upon treatment with tunicamycin, an inducer of misfolded proteins (Fig. 6 I). Tunicamycin or TAK-243, an inhibitor of the ubiquitin-activating enzyme UBA1, had no effect on endogenous MIDN protein levels in B cells (Fig. 6, J and K). However, MG132, a proteasome inhibitor (Goldberg, 2012), increased MIDN levels in B cells, specifically inducing the smaller of the two detected isoforms while the larger isoform was similar in abundance before and after MG132 treatment (Fig. 6, J and K).

**Figure S5.**
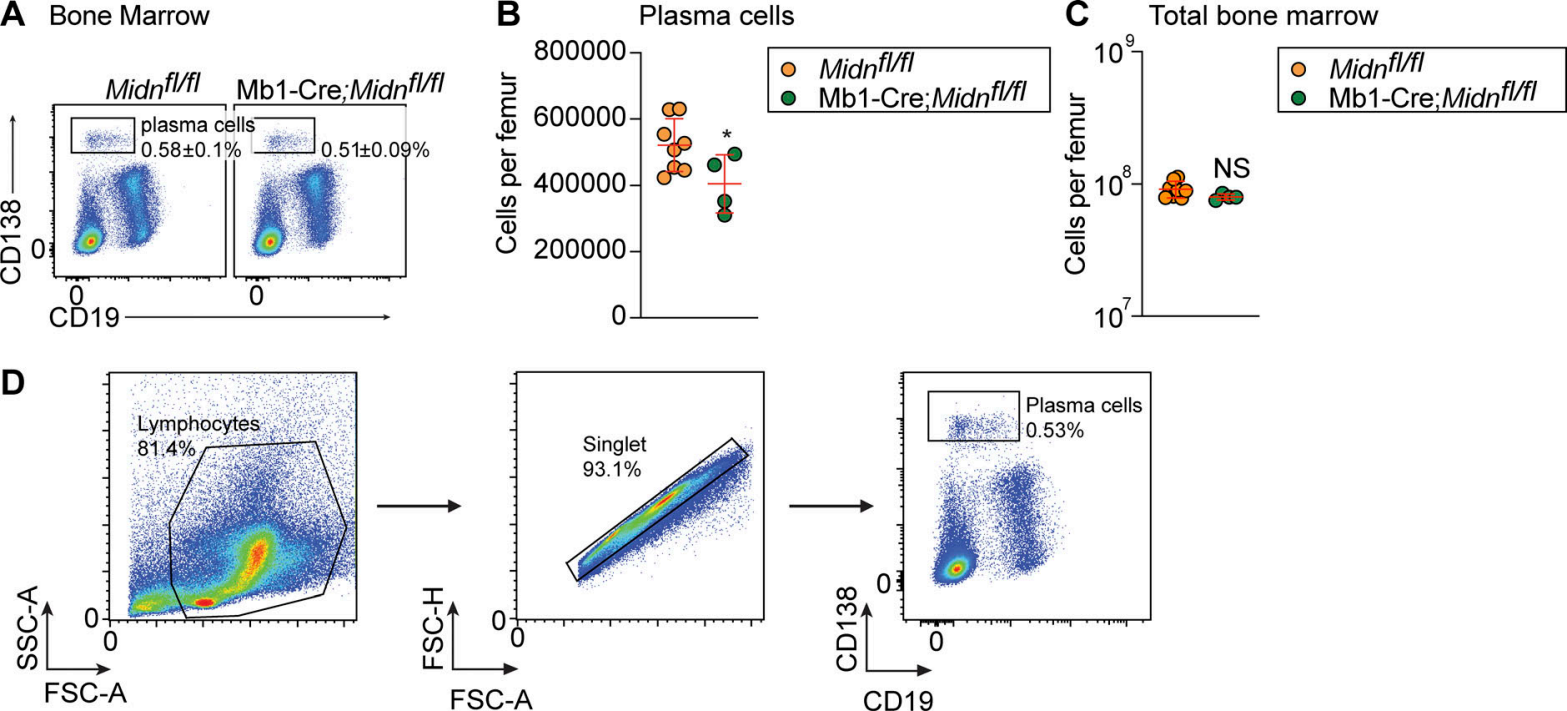
**Plasma cell production after immunization with the TD antigen β-galactosidase in Mb1-Cre;*Midn***^***fl/fl***^ **mice. (A and B)** Representative flow cytometry plots (A) and numbers (B) of plasma cells in the bone marrow of 8-wk-old Mb1-Cre;*Midn*^*fl/fl*^ and *Midn*^*fl/fl*^ littermates after immunization with β-galactosidase, delivered in a rSFV vector (rSFV-βgal) (Hidmark et al., 2006) (*n* = 4 Mb1-Cre;*Midn*^*fl/fl*^ and 8 *Midn*^*fl/fl*^ littermates). **(C)** Total numbers of bone marrow cells per femur in 8-wk-old Mb1-Cre;*Midn*^*fl/fl*^ and *Midn*^*fl/fl*^ littermates (*n* = 4 Mb1-Cre;*Midn*^*fl/fl*^ and 8 *Midn*^*fl/fl*^ littermates). **(D)** Gating strategy for plasma cells. Heterozygous carriers of the Mb1-Cre transgene were used in A–C. Data are representative of two independent experiments. Data points represent individual mice (B and C). Error bars indicate SD. P values were determined by Student’s *t* test (B and C). * P < 0.05; NS, not significant.

In addition to mediating the transcriptional response to unfolded proteins, XBP1s promotes plasma cell differentiation, and, B cell–specific XBP1s transgenic overexpression drives transformation to multiple myeloma (Carrasco et al., 2007; Reimold et al., 2001). Since XBP1s was elevated in *Midn*^*KD/KD*^ B cells, we checked whether plasma cells might be increased as a result of *Midn* deletion. We immunized mice with the TD antigen β-galactosidase to induce expansion of plasma cells. We detected similar frequencies of plasma cells in *Midn*^*fl/fl*^ mice and Mb1-Cre;*Midn*^*fl/fl*^ mice following immunization with β-galactosidase (Fig. S5), indicating that *Midn* deficiency leading to upregulation of XBP1s does not promote plasma cell expansion.

### Acute deletion of *Midn* suppressed established lymphoproliferative disease

**Figure 7.**
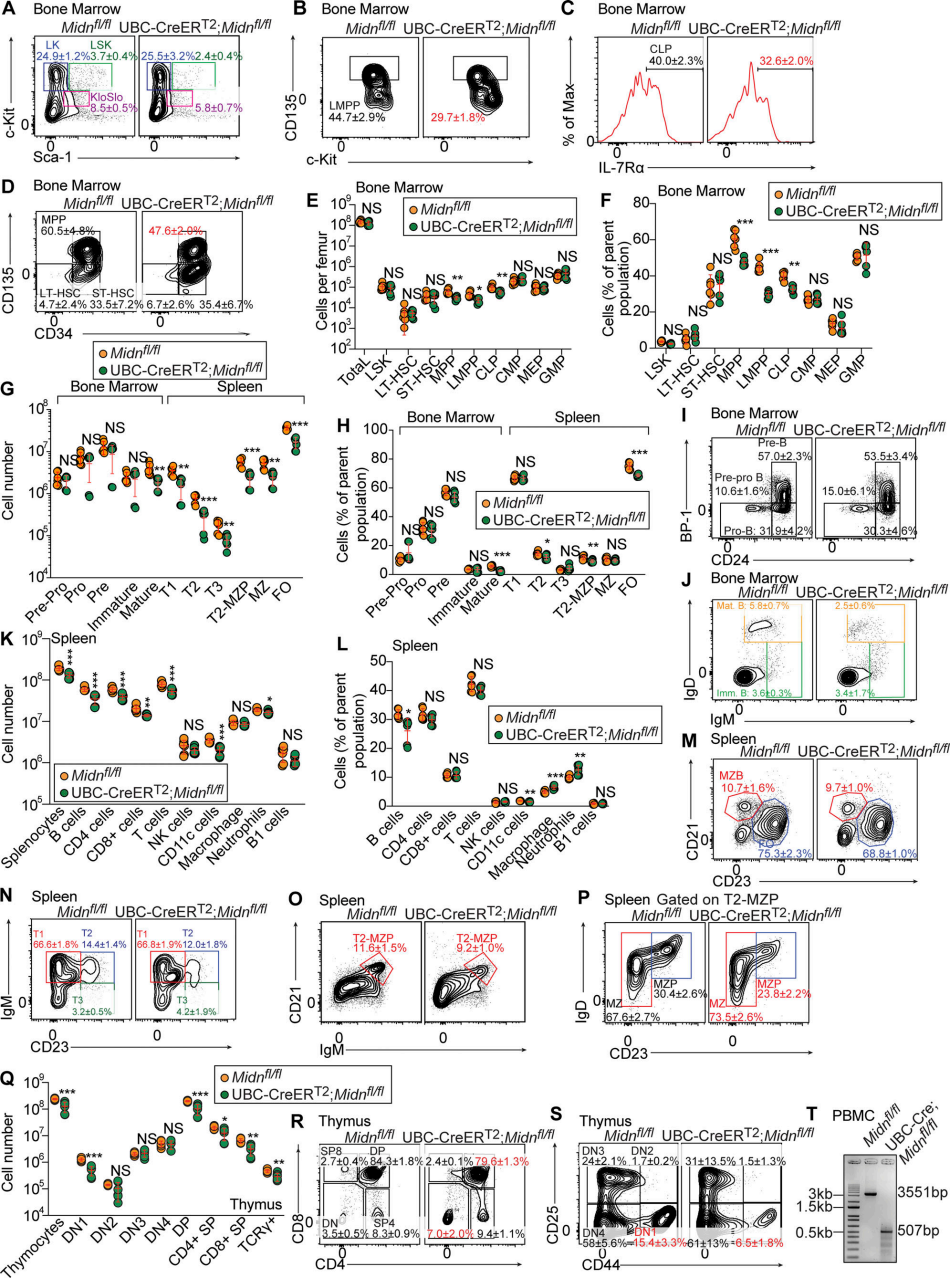
**Analysis of HSPCs, B cell development, and T cell development in adult mice after acute deletion of *Midn*. (A–D)** Representative flow cytometry plots of HSPC populations in 10-wk-old UBC-Cre-ER^T2^;*Midn*^*fl/fl*^ and *Midn*^*fl/fl*^ littermates (*n* = 6 mice/genotype) on day 13 after tamoxifen treatment. **(E and F)** Numbers (E) and frequencies (F) of bone marrow HSPC populations in 10-wk-old UBC-Cre-ER^T2^;*Midn*^*fl/fl*^ and *Midn*^*fl/fl*^ littermates (*n* = 6 mice/genotype) on day 13 after tamoxifen treatment. **(G and H)** Numbers (G) and frequencies (H) of B cell subpopulations in the bone marrow and spleen of 10-wk-old UBC-Cre-ER^T2^;*Midn*^*fl/fl*^ and *Midn*^*fl/fl*^ littermates (*n* = 6 mice/genotype) on day 13 after tamoxifen treatment. **(I and J)** Representative flow cytometry plots showing B cell development in the bone marrow of 10-wk-old UBC-Cre-ER^T2^;*Midn*^*fl/fl*^ and *Midn*^*fl/fl*^ littermates (*n* = 6 mice/genotype) on day 13 after tamoxifen treatment. **(K and L)** Numbers (K) and frequencies (L) of splenocytes and the indicated immune cell populations in the spleen of 10-wk-old UBC-Cre-ER^T2^;*Midn*^*fl/fl*^ and *Midn*^*fl/fl*^ littermates (*n* = 6 mice/genotype) on day 13 after tamoxifen treatment. **(M–P)** Representative flow cytometry plots showing B cell development in the spleen of 10-wk-old UBC-Cre-ER^T2^;*Midn*^*fl/fl*^ and *Midn*^*fl/fl*^ littermates (*n* = 6 mice/genotype) on day 13 after tamoxifen treatment. **(Q)** Numbers of thymocyte subpopulations in the thymus of 10-wk-old UBC-Cre-ER^T2^;*Midn*^*fl/fl*^ and *Midn*^*fl/fl*^ littermates (*n* = 6 mice/genotype) on day 13 after tamoxifen treatment. **(R and S)** Representative flow cytometry plots showing T cell development in the thymus of 10-wk-old UBC-Cre-ER^T2^;*Midn*^*fl/fl*^, and *Midn*^*fl/fl*^ littermates (*n* = 6 mice/genotype) on day 13 after tamoxifen treatment. **(T)** PCR analysis of genomic DNA isolated from tamoxifen-treated UBC-Cre-ER^T2^;*Midn*^*fl/fl*^ and *Midn*^*fl/fl*^ mice. *Midn*^*fl*^ allele product: 3,551 bp before or 507 bp after Cre-mediated deletion. Flow cytometry gating strategies to detect B cells, T cells, CD4 T cells, CD8 T cells, NK cells, CD11c^+^ cells, macrophages, neutrophils, and B-1 B cells in blood or spleen are shown in Fig. S17A of Zhong et al. (2023). Flow cytometry gating strategies to analyze B cell development in the bone marrow and spleen are shown in Fig. S3 of Choi et al. (2020). Flow cytometry gating strategies to analyze HSPC populations, except LMPP, are shown in Fig. S1 of Choi et al. (2020). Flow cytometry gating strategies to analyze LMPP are shown in Fig. S2 A of this paper. Data are representative of two independent experiments. Data points represent individual mice (E–H, K, L, and Q). Error bars indicate SD. P values were determined by Student’s *t* test (E–H, K, L, and Q). * P < 0.05; ** P < 0.01; *** P < 0.001; NS, not significant. Source data are available for this figure: SourceData F7.

We crossed *Midn*^*fl/fl*^ mice to the B6 congenic UBC-Cre-ER^T2^ strain, which expresses a tamoxifen-inducible Cre transgene throughout the body (Ruzankina et al., 2007). In UBC-Cre-ER^T2^;*Midn*^*fl/fl*^ mice, tamoxifen-induced Cre activity results in deletion of *Midn* in all tissues. On day 13 after tamoxifen injection, multipotent progenitors (MPP), lymphoid-primed MPPs (LMPP), and common lymphoid progenitors (CLP) were reduced in number and frequency in the bone marrow of UBC-Cre-ER^T2^;*Midn*^*fl/fl*^ mice, while all other HSPC populations were unaffected by *Midn* deletion (Fig. 7, A–F). In the bone marrow, mature B cells were reduced in number and frequency, but B cell progenitor and immature B cell numbers and frequencies were normal in tamoxifen-treated UBC-Cre-ER^T2^;*Midn*^*fl/fl*^ mice (Fig. 7, G–J).

Total splenocytes and most splenic lymphoid and myeloid cell population numbers were reduced after acute deletion of *Midn*; but only B cell and CD11c^+^ cell frequencies were reduced (Fig. 7, K and L). Among B cells that entered the spleen, populations of transitional, MZ, and follicular B cells were reduced in number (Fig. 7 G), but frequencies of these populations were only mildly or not affected in UBC-Cre-ER^T2^;*Midn*^*fl/fl*^ mice (Fig. 7, H and M–P). Splenic B-1 B cell numbers and frequencies were normal in UBC-Cre-ER^T2^;*Midn*^*fl/fl*^ mice, in contrast to *Midn*^*KD/KD*^ mice (Fig. 7, K and L). Since B-1 B cells are predominantly derived from embryonic, fetal, and neonatal precursors, and in adulthood are maintained or replenished by self-renewal (Baumgarth, 2017; Ghosn et al., 2011), the normal numbers and frequencies of B-1 B cells after acute deletion of *Midn* in adult mice suggest that B-1 B cell survival and/or self-renewal may not require MIDN, but MIDN is required for their de novo development. Another possibility is that the time point after induction of *Midn* deletion is too early to detect a change in overall B-1 B cell numbers.

T cell development in the thymus of UBC-Cre-ER^T2^;*Midn*^*fl/fl*^ mice was similar to that observed in *Midn*^*KD/KD*^ mice, resulting in similar deficits in thymocyte numbers (Fig. 7, Q–S). In UBC-Cre-ER^T2^;*Midn*^*fl/fl*^ mice, there was additionally a partial block at the DN to DP transition (Fig. 7 R).

Overall, lymphoid progenitor stages were detrimentally affected in UBC-Cre-ER^T2^;*Midn*^*fl/fl*^ mice but not in *Midn*^*KD/KD*^ mice. In contrast, B cell development in the bone marrow and spleen was less impaired in UBC-Cre-ER^T2^;*Midn*^*fl/fl*^ mice than in *Midn*^*KD/KD*^ mice (Fig. 7 T).

**Figure 8.**
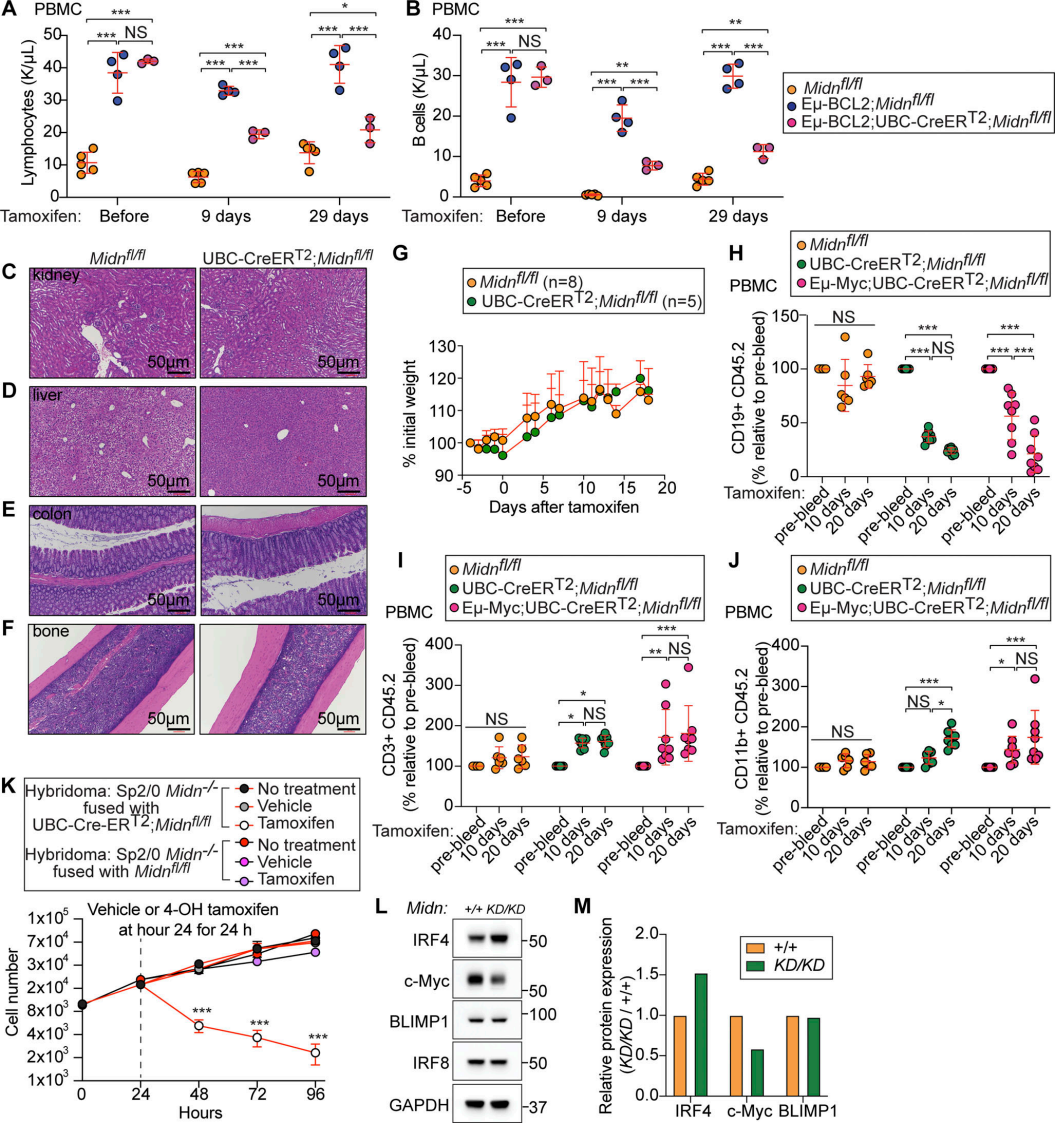
**Acute deletion of *Midn* suppressed established lymphoproliferative disease. (A and B)** Numbers of lymphocytes (A) and B cells (B) in the blood of 10-wk-old WT mice, Eμ-BCL2 mice, or Eμ-BCL2;UBC-Cre-ER^T2^;*Midn*^*fl/fl*^ mice before tamoxifen treatment or on the indicated days after tamoxifen treatment (*n* = 3 Eμ-BCL2;UBC-Cre-ER^T2^;*Midn*^*fl/fl*^, 4 Eμ-BCL2;*Midn*^*fl/fl*^, 5 *Midn*^*fl/fl*^ littermates). **(C–F)** Representative images of H&E stained tissue sections from kidney (C), liver (D), colon (E), and bone (F) of 10-wk-old *Midn*^*fl/fl*^ and UBC-Cre-ER^T2^;*Midn*^*fl/fl*^ littermates 10 days after tamoxifen treatment. **(G)** Body weights of 10-wk-old *Midn*^*fl/fl*^ (*n* = 8 mice) and UBC-Cre-ER^T2^;*Midn*^*fl/fl*^ littermates (*n* = 5 mice) on the indicated days after tamoxifen treatment. **(H–J)** 12 wk after transplantation of bone marrow from mice of the indicated genotypes into lethally irradiated CD45.1 WT recipient mice, peripheral blood CD19^+^ cells (B cells) (H), CD3^+^ cells (T cells) (I), and CD11b^+^ cells (J) of donor origin (CD45.2) were analyzed by flow cytometry before (pre-bleed) and 10 and 20 days after tamoxifen treatment. Cell counts are normalized to pre-bleed cell counts (*n* = 8 Eμ-Myc;UBC-Cre-ER^T2^;*Midn*^*fl/fl*^ recipients, 8 Eμ-Myc;*Midn*^*fl/fl*^ recipients, 6 *Midn*^*fl/fl*^ recipients). **(K)** Growth curve of Sp2/0 *Midn*^*−/−*^ cells rescued by fusion with B cells from UBC-Cre-ER^T2^;*Midn*^*fl/fl*^ or *Midn*^*fl/fl*^ mice (which express MIDN) (*n* = 4 wells/genotype or treatment). *Midn* was then deleted in the fused cells by tamoxifen treatment for 24 h beginning at hour 24 in culture. **(L and M)** Immunoblot analysis (L) and quantitation (M) of the indicated proteins in lysates of WT and *Midn*^*KD/KD*^ B cells. Data are representative of two independent experiments. Data points represent individual mice (A and B) or recipient mice (H–J). Error bars indicate SD. P values were determined by two-way analysis of variance (ANOVA) with Dunnett’s multiple comparisons (A, B, and G–K). * P < 0.05; ** P < 0.01; *** P < 0.001; NS, not significant. Source data are available for this figure: SourceData F8.

We crossed UBC-Cre-ER^T2^;*Midn*^*fl/fl*^ mice to Eμ-BCL2 mice. The elevated lymphocyte and B cell counts observed in Eμ-BCL2 mice were greatly reduced by acute deletion of *Midn* (Fig. 8, A and B). In contrast, histological analyses showed normal tissue structure and gross cellular morphology in the bone marrow, liver, kidney, and intestine of UBC-Cre-ER^T2^;*Midn*^*fl/fl*^ mice after tamoxifen administration (Fig. 8, C–F). UBC-Cre-ER^T2^;*Midn*^*fl/fl*^ mice also maintained body weight after *Midn* deletion, suggesting the absence of pathological effects of acute *Midn* deletion in postnatal animals (Fig. 8 G).

To test the effect of acute *Midn* deletion on Eμ-Myc driven leukemia, we transplanted bone marrow from Eμ-Myc transgenic or non-transgenic UBC-Cre-ER^T2^;*Midn*^*fl/fl*^ mice (CD45.2) to lethally irradiated WT recipients (CD45.1). After engraftment, we analyzed CD45.2 B cells, T cells, and CD11b^+^ cells in the blood by flow cytometry before and after tamoxifen treatment. *Midn* deletion induced the elimination of both leukemic Eμ-Myc transgenic B cells and non-leukemic B cells lacking the Eμ-Myc transgene (Fig. 8 H). However, T cells and CD11b^+^ cells maintained their numbers after *Midn* deletion (Fig. 8, I and J).

We also tested the effect of *Midn* deletion in Sp2/0-Ag14 (Sp2/0) hybridoma cells (a myeloma–B cell fusion). We first knocked out *Midn* in Sp2/0 cells by CRISPR targeting. Sp2/0 *Midn*^*−/−*^ cells were growth arrested, suggesting that they required MIDN to proliferate. We then fused Sp2/0 *Midn*^*−/−*^ cells with B cells from UBC-Cre-ER^T2^;*Midn*^*fl/fl*^ mice, which express MIDN in the absence of tamoxifen, rescuing the growth defect (Fig. 8 K). When we treated these fused Sp2/0 *Midn*^*−/−*^;UBC-Cre-ER^T2^;*Midn*^*fl/fl*^ cells with tamoxifen, we observed rapid elimination of viable Sp2/0 cells (Fig. 8 K), demonstrating that acute deletion of MIDN was lethal to Sp2/0 cells.

Based upon the finding that MIDN promoted proteasome-mediated degradation of IRF4 (Gu et al., 2023), and reports that IRF4 promoted c-*Myc* expression necessary for myeloma cell survival (Shaffer et al., 2008), we analyzed IRF4 and c-MYC levels in *Midn*^*KD/KD*^ splenic B cells by immunoblot. IRF4 levels were elevated but c-MYC levels were reduced in *Midn*^*KD/KD*^ cells with respect to levels in WT cells (Fig. 8, L and M). Since *Midn*^*KD/KD*^ mice had normal frequencies of plasma cells and homozygosity for the *Midn*^*KD*^ allele suppressed the B cell cancers tested here, IRF4 is unlikely to be a driver of lymphoproliferation in these settings. Rather, suppression of these malignancies may involve an increase in IRF4 as previously reported (Pathak et al., 2011).

## Discussion

This work addresses a longstanding question concerning the function of MIDN in vivo. We analyzed the phenotypes of viable mice with ENU-induced point mutations in *Midn*, with a targeted mutation resulting in deletion of four of six MIDN protein isoforms, with tamoxifen-inducible deletion of all MIDN isoforms, and with B cell–specific MIDN deletion. We demonstrated that outside of its essential function in organism development, MIDN is of particular importance in B cell development and function. Supporting this claim is the specific impairment of *Midn*^*KD/KD*^ HSC in reconstituting B cells in mixed bone marrow chimeras, while other *Midn*^*KD/KD*^ lymphoid and myeloid lineages repopulated as well as WT cells. Importantly, this finding translated to a unique susceptibility of Eμ-Myc–driven leukemia and lymphoma to death upon *Midn* deletion in vivo. The sensitivity of Eμ-Myc transgenic B cells to loss of MIDN likely results from the known dependence of Myc-driven lymphoma on the UPR for survival in the context of upregulated protein synthesis (Barna et al., 2008; Ruggero, 2009; Zhang et al., 2020). In the absence of MIDN, proteostasis mediated by the action of proteasomes is impaired and induction of an apoptotic UPR probably occurs. We noted that germline mutant *Midn*^*KD/KD*^ mice had reduced bone, muscle, and total body mass, but adult mice maintained their body weight and had histologically normal bone marrow, liver, kidney, and intestine after acute deletion of *Midn*. These findings indicate a role for MIDN in development and suggest that targeting MIDN in adulthood may be a safe and effective treatment for B cell cancers. However, it is possible that examining adult mice later after *Midn* deletion may reveal effects more similar to those observed in *Midn*^*KD/KD*^ mice.

We observed differences between the hematopoietic phenotypes of germline mutant *Midn*^*KD/KD*^ mice, B cell–specific *Midn* null mice (Mb1-Cre;*Midn*^*fl/fl*^), and bone marrow chimeric mice that point to both B cell–extrinsic and hematopoietic-extrinsic functions of MIDN. For example, *Midn*^*KD/KD*^ NK cell and macrophage numbers were normal in bone marrow chimeras but reduced in germline mutant mice, suggesting that MIDN has hematopoietic-extrinsic effects that support the development of these cells. Differences between Mb1-Cre;*Midn*^*fl/fl*^ and *Midn*^*KD/KD*^ B cell progenitor and immature B cell numbers suggest that B cell–extrinsic effects may contribute to the greater reductions in absolute numbers of B cell populations in *Midn*^*KD/KD*^ mice. Intriguingly, we observed greater T cell reconstitution by *Midn*^*KD/KD*^ bone marrow than by WT bone marrow, suggesting that reduced MIDN function may confer a cell-intrinsic competitive advantage for HSC development to the T cell lineage.

We showed that lysates of *Midn*^*KD/KD*^ tissues had reduced proteasome activity compared with the corresponding WT tissues and that purified MIDN directly stimulated proteasome activity in vitro. These findings raise the possibility that MIDN acts as a component of proteasomes in B cells and other tissues that serve to generally boost proteasome activity, consistent with the reported stable association between MIDN and proteasomes (Gu et al., 2023). However, the physical mechanism by which MIDN increases proteasome activity remains unknown. It has been shown that other UBL domain–containing (UBLD) proteins (HR23B, USP14, UBLCP1, Parkin, UBL4A, UBQLN1) or UBL domains by themselves allosterically increase the degradative activity of the proteasome even in the absence of conjugation to ubiquitin (Collins and Goldberg, 2020; Kim and Goldberg, 2018; Yu et al., 2016; Zhang et al., 2022). MIDN, however, was reported to interact with proteasomes using its C-terminal α-helix rather than its UBL domain (Gu et al., 2023), suggesting a different mechanism. Both our proteasome activity data and assays of MIDN-dependent substrate degradation (Gu et al., 2023) demonstrated the importance of the UBL domain for MIDN function. Yet rescue of the pre-weaning lethality of *Midn* null mice, likely by the presence of the 358-aa and/or 401-aa MIDN isoforms in *Midn*^*KD/KD*^ mice (see Fig. S1, B–D), indicates that the function of MIDN lacking the UBL is sufficient to support life.

Beyond the nature of the MIDN-proteasome interaction, we do not yet understand which proteins depend on MIDN to regulate their cellular abundance in support of B cell development and function. Transcription factors, some of which are specific to the immune system, were enriched among proteins reported to require MIDN for degradation (Gu et al., 2023). Consistent with that finding, in HEK293T cells, MIDN was predominantly localized in the nucleus, where it increased in abundance after proteasome inhibitor (MG132) treatment (Gu et al., 2023). This contrasts with our data showing that MIDN was predominantly localized in the cytosol in splenic B cells; whether this reflects a different set of dependent proteins remains unknown. The use of different cell types, the induction of ER stress by treatment with MG132 versus no treatment, and the quiescent state of unstimulated B cells versus the proliferative state of HEK293T cells may account for the difference in localization.

Another question concerns the stimuli that activate or upregulate MIDN in vivo. We showed that two isoforms of MIDN exist in splenic B cells. The larger isoform predominated in unstimulated B cells, but MG132 treatment induced the smaller isoform without altering the abundance of the larger one. Interestingly, two forms of MIDN were also detected in HEK293T cells (Gu et al., 2023). This suggests distinct functions might be carried out by the two isoforms, the sequences of which are not yet known. Is the large isoform a constitutive part of some proteasomes in B cells? Does the small isoform regulate different proteins than the large isoform? What other stimuli induce the small isoform (or the large one)? These and other questions about MIDN await answers that will increase our understanding of normal and malignant B cell biology and the regulation of proteostasis.

## Materials and methods

### Mice

8- to 10-wk-old pure C57BL/6J male mice purchased from The Jackson Laboratory were mutagenized with ENU as described previously (Wang et al., 2015). Mutagenized G0 males were bred to C57BL/6J females, and the resulting G1 males were crossed to C57BL/6J females to produce G2 mice. G2 females were backcrossed to their G1 sires to yield G3 mice, which were screened for phenotypes at 7–16 wk of age. Whole-exome sequencing and mapping were performed as described (Wang et al., 2015).

Homozygous *Midn* knockout mice (*Midn*^*em1(IMPC)Bay*^) are described at https://www.mousephenotype.org/data/genes/MGI:1890222. To generate *Midn*^*KD/KD*^ mice, female C57BL/6J mice were superovulated and mated with C57BL/6J male mice as described before (Choi et al., 2019; Zhong et al., 2020, 2022). Fertilized eggs were collected and in vitro transcribed Cas9 mRNA (50 ng/μl), and *Midn* small base-pairing guide RNA (50 ng/μl; 5′-ATG​AGG​CCA​GCT​TCC​ACC​GT-3′) was injected into the cytoplasm or pronucleus of the embryos. The injected embryos were cultured in M16 medium (Sigma-Aldrich) and two-cell stage embryos were transferred into the ampulla of the oviduct (10–20 embryos per oviduct) of pseudopregnant Hsd:ICR (CD-1) (Harlan Laboratories) females (Choi et al., 2019; Zhong et al., 2020, 2022).

The analysis of founder *Midn*^*KD/KD*^ mice showed a 19-bp deletion (bracketed sequence deleted: 5′-AGT​TGA​CGC​T[CGTGCCCACGGTGGAAGCT]GGC​CTC​ATG​GT-3′) in transcripts NM_021565.2, NM_001388478.1, NM_001347117.1, NM_001305798.1, NM_001388477.1, and XM_006513913.4 within the third exon, or transcripts NM_001305799.1, NM_001388476.1, NM_001388475.1, XM_036155903.1, and XM_030245189.2 within the second exon. The allele is predicted to result in a frameshifted protein product beginning after amino acid 99 of the protein, which is normally 508, 507, 465, or 464 amino acids in length, and terminating after the inclusion of 19 aberrant amino acids. However, XP_036011796.1 and XP_030101049.1, which are 401 and 358 amino acids in length, respectively, were intact.

Female C57BL/6J mice were superovulated and mated with C57BL/6J male mice as described before (Choi et al., 2019; Zhong et al., 2020, 2022). To generate mice carrying 3x-FLAG or 2x-HA at the C terminus of MIDN protein, in vitro transcribed Cas9 mRNA (50 ng/μl), *Midn* small base-pairing guide RNA (50 ng/μl; 5′-CCG​AGT​TTG​TGG​TGG​CTT​AA-3′), and Ultramar template DNA were injected into the cytoplasm or pronucleus of the embryos. *Midn*^*fl/fl*^ mice carrying *lox*P sites flanking *Midn* exons 4, 5, and 6 (containing 184 bp coding sequence) were obtained by injecting in vitro transcribed Cas9 mRNA (50 ng/μl), *Midn* small base-pairing guide RNA (50 ng/μl; up: 5′-CCC​ACT​GCC​CCT​AGC​ACG​CA-3′ and down: 5′-TCC​AAG​CCT​CCT​TGG​GGT​TT-3′), and Ultramar template DNA into the cytoplasm or pronucleus of the embryos. For genotyping of 3x-FLAG, 2x-HA, or *loxP* insertion, the PCR and sequencing primers are listed in Table S1.

C57BL/6J (#000664), C57BL/6.SJL (CD45.1) (#002014), *Rag2*^*−/−*^ (#008449), B6.Cg-*Ndor1*^*Tg(UBC-cre/ERT2)1Ejb*^/2J (UBC-Cre-ER^T2^) (#007001), B6.C(Cg)-*Cd79a*^*tm1(cre)Reth*^/EhobJ (Mb1-Cre) (#020505), B6.Cg-Tg(BCL2)22Wehi/J (Eμ-BCL2) (#002319), and B6.Cg-Tg(IghMyc)22Bri/J (Eμ-Myc) (#002728) mice were purchased from The Jackson Laboratory. The following strains were generated by intercrossing mouse strains: (1) UBC-Cre-ER^T2^;*Midn*^*fl/fl*^, (2) Mb1-Cre;*Midn*^*fl/fl*^, (3) *Midn*^*Flag/Flag*^, (4) Eμ-BCL2;Mb1-Cre;*Midn*^*fl/fl*^, (5) Eμ-myc;Mb1-Cre;*Midn*^*fl/fl*^, (6) Eμ-BCL2;UBC-Cre-ER^T2^;*Midn*^*fl/fl*^, and (7) CD45.1;*Rag2*^*−/−*^. All purchased mouse strains were on the C57BL/6J background as specified on the JAX website; all mouse strains we generated using pure C57BL/6J mice (mutagenesis) or embryos (CRISPR/Cas9 engineering). Mice of both sexes were used in experiments, and data were combined for analyses. Conventionally, reared mice were housed in specific pathogen–free conditions, 12 h light/12 h dark cycle, 20–26°C ambient temperature, and 30–70% humidity at the University of Texas (UT) Southwestern Medical Center. Animal work described in this manuscript has been approved and conducted under the oversight of the UT Southwestern Institutional Animal Care and Use Committee.

### Flow cytometry analysis of immune cells in blood, bone marrow, spleen, and thymus

Most flow cytometry gating strategies were previously published and the precise locations where they can be found are indicated in the figure legend of each figure where flow cytometry data are shown. The gating strategy for LMPP cells is shown in Fig. S2 A. The gating strategy for plasma cells is shown in Fig. S5 D.

Peripheral blood was collected and treated as described before (Choi et al., 2019; Zhong et al., 2020, 2022). Briefly, red blood cells (RBCs) were lysed with hypotonic buffer (eBioscience), and RBC-depleted samples were stained for 1 h at 4°C in 100 μl of a cocktail of fluorescence-conjugated antibodies (diluted 1:200) to 15 cell surface markers encompassing the major immune lineages: B220 (clone RA3-6B2; BD), CD19 (clone 1D3; BD), IgM (clone R6-60.2; BD), IgD (clone 11-26c.2a; BioLegend), CD3ε (clone 145-2C11; BD), CD4 (clone RM4-5; BD), CD8α (clone 53-6.7; BioLegend), CD11b (clone M1/70; BioLegend), CD11c (clone HL3; BD), F4/80 (clone BM8.1; Tonbo), CD44 (clone 1M7; BD), CD62L (clone MEL-14; Tonbo), CD5 (clone 53-7.3; BD), CD43 (clone S7; BD), NK 1.1 (clone OK136; BioLegend), and 1:200 Fc block (clone 2.4G2; Tonbo). Flow cytometry data were collected on a BD LSR Fortessa, and the proportions of immune cell populations in each G3 mouse were analyzed with FlowJo software. The resulting screening data were used for automated mapping of causative alleles (Wang et al., 2015).

To stain the B cell and T cell subsets, bone marrow cells, splenocytes, or thymocytes were isolated, and RBC lysis buffer was added to remove RBCs. Cells were stained at 1:200 antibody dilution in the presence of anti-mouse CD16/32 antibody (clone 2.4G2; Tonbo) and different antibody cocktails: B cells in bone marrow (IgM [clone R6-60.2; BD], IgD [clone 11-26c.2a; BioLegend], B220 [clone RA3-6B2; BD], CD43 [clone S7; BD], CD24 [clone M1/69; BioLegend], Ly-51 [clone BP-1; BD], CD19 [clone 1D3; BD]); splenic B cells (CD4 [clone RM4-5; BD], IgM [clone R6-60.2; BD], IgD [clone 11-26c.2a; BioLegend], B220 [clone RA3-6B2; BD], CD23 [clone B3B4; BD], CD21/CD35 [clone 7E9; BioLegend], CD93 [clone AA4.1; BD]); and thymocytes (CD25 [clone 3C7; BioLegend], CD44 [clone 1M7; BD], CD4 [clone RM4-5; BD], CD8α [clone 53-6.7; BioLegend], CD69 [clone H1.2F3; eBioscience], CD5 [clone 53-7.3; BD], CD117 [c-kit] [clone 2B8; BioLegend], TCR-β [clone H57-597; BD], PD-1 [clone 29F.1A12; eBioscience], CD197 [CCR7] [clone 4B12; BioLegend], Helios [clone 22F6; BioLegend]) (Baran-Gale et al., 2020; Choi et al., 2019; Yui et al., 2010; Zhong et al., 2020, 2022). HSPC were stained with Alexa Fluor 700 anti-mouse lineage cocktail (clones 17A2/RB6-8C5/RA3-6B2/Ter-119/M1/70; BioLegend), CD34 (clone RAM34; eBioscience), CD135 (clone A2F10; BioLegend), CD16/CD32 (clone 93; eBioscience), CD127 (clone SB/199; BD), Ly-6A/E (Sca-1) (clone D7; BioLegend), and CD117 (c-kit) (clone 2B8; BioLegend) (Choi et al., 2019; Zhong et al., 2020, 2022).

For plasma cell detection, 12-wk-old *Midn*^*fl/fl*^ or Mb1-cre;*Midn*^*fl/fl*^ mice were intramuscularly immunized with 2 million recombinant Semliki Forest virus (rSFV) particles on day 0 (Choi et al., 2019; Hidmark et al., 2006). Mice were boosted 1 mo after the first injection. 3 mo after the prime injection, bone marrow was flushed for analysis of plasma cells using antibodies to CD138 (clone 281-2; BioLegend), B220 (clone RA3-6B2; BD), and CD19 (clone 1D3; BD).

### Skeletal phenotype analyses

Skeletal phenotype analyses were performed as previously described (Rios et al., 2021). Briefly, dual-energy xx-ray absorptiometry was performed in anesthetized mice using an UltraFocusDXA instrument (Hologic Inc). Femur bone density and whole-body (less head) lean mass were calculated using the region-of-interest tool within the Hologic software. As described previously (Rios et al., 2021), residual differences in each phenotype measurement compared to age- and sex-matched normative values were plotted.

### Bone marrow chimeras

Bone marrow transplantation was performed as previously described (Choi et al., 2019; Zhong et al., 2020, 2022). Recipient mice were lethally irradiated (7 Gy or 700 rads) by an x-ray irradiator twice at a 5-h interval. Femurs from donor mice were flushed with PBS using a 25G needle. To remove bits of bone, the marrow was homogenized, and the solution was filtered through a sterile 40-µm nylon cell strainer (BD Biosciences). RBC-depleted cells were pelleted and resuspended in 1 ml PBS and kept on ice. Bone marrow cells from donors were transferred into the irradiated recipients through retro-orbital injection. For 4 wk after engraftment, mice were maintained on an antibiotic. 12 wk after bone marrow transplantation, peripheral blood was sampled, and immune cells were assessed with flow cytometry as above using fluorescence-conjugated antibodies. To distinguish CD45 congenic markers, antibodies against CD45.1 (cloneA10; BioLegend) and CD45.2 (clone 104; BioLegend) were used.

### Immunization and enzyme-linked immunosorbent assay (ELISA) analysis

Immunization and ELISA were performed as previously described (Choi et al., 2019; Zhong et al., 2020, 2022). Briefly, 12-wk-old mice were intramuscularly immunized with ovalbumin (200 μg; Invivogen) + alum (20 μg; Invivogen) as adjuvant on day 0 and intraperitoneally immunized with NP_50_-AECM-Ficoll (50 μg; Biosearch Technologies) on day 8 as previously described. Blood was collected on day 6 after NP-Ficoll immunization in minicollect tubes (Mercedes Scientific) and centrifuged at 1,500 × *g* to separate serum for analysis of antigen-specific IgG or IgM concentration by ELISA.

For ELISA analysis of antigen-specific IgG and IgM responses, Nunc MaxiSorp flat-bottom 96-well microplates (Thermo Fisher Scientific) were coated with 5 μg/ml soluble ovalbumin (Invivogen) or NP8-BSA (Biosearch Technologies) and incubated at 4°C overnight. Plates were washed four times with washing buffer (0.05% [vol/vol] Tween-20 in PBS) using a BioTek microplate washer and then blocked with 1% (vol/vol) BSA for 1 h at room temperature. Serum samples were serially diluted in 1% (vol/vol) BSA and then the 1:50 and 1:150 dilutions were added to the prepared ELISA plates. After a 2 h incubation, the plates were washed eight times with washing buffer and then incubated with HRP-conjugated goat anti-mouse IgG or IgM for 1 h at room temperature. Plates were washed eight times with washing buffer and then developed with SureBlue TMB Microwell Peroxidase Substrate and TMB Stop Solution (KPL). Absorbance was measured at 450 nm on a Synergy Neo2 Plate Reader (BioTek). Basal levels of anti-ova IgG and anti-NP IgM were determined using preimmune serum. All ELISA data shown represent the 1:150 serum dilution (Choi et al., 2019; Zhong et al., 2020, 2022).

### In vitro stimulation of B cells

Splenic B cells were negatively enriched using the EasySep Mouse Pan-B cell Isolation Kit (StemCell Technologies). For the in vitro stimulation assay, 3 × 10^6^ B cells were treated for 6 h at 37°C with tunicamycin (33.3 μg/ml) (#12819S; CST), MG132 (10 μM) (#M7449; Sigma-Aldrich), and TAK-243 (500 nM) (# HY-100487; MedChemExpress). Then, the cells were washed twice in sterile PBS to remove residual proteins in the culture media. Proteins levels were measured using immunoblot.

### Cell culture, transfection, co-IP, and immunoblotting

HEK293T cells were grown at 37°C in DMEM (Life Technologies)/10% (vol/vol) FBS (Gibco)/1% antibiotics (Life Technologies) in 5% CO_2_. Transfection of plasmids was carried out using Lipofectamine 2000 (Life Technologies) according to the manufacturer’s instructions. 36–48 h after transfection, cells were harvested in NP-40 lysis buffer (20 mM Tris–Cl, pH 7.5, 150 mM NaCl, 1 mM EDTA, 1 mM EGTA, 1% [vol/vol] NP-40, 2.5 mM Na_4_P_2_O_7_, 1 mM C_3_H_9_O_6_P, 1 mM Na_3_VO_4_, and protease inhibitors) for 45 min at 4°C. Co-IP assays were performed using cell extracts from HEK293T cells overexpressing MIDN^WT^, PSMC2, PSMC4, PSMC5, or PSMD11 proteins in separate cultures. Extracts of cells expressing the proteins of interest were mixed. Proteins were immunoprecipitated by anti-Flag M2 affinity gel (Sigma-Aldrich). Captured proteins were eluted by PBS with 30 μl of 200 μg/ml 3xFlag peptides (Sigma-Aldrich), and 8 μl of sample buffer was mixed with 0.1% bromphenol blue, heated to 95°C for 3 min, centrifuged at 12,000 × *g* for 1 min, and the supernatants were loaded onto SDS-PAGE for immunoblotting analysis by anti-Flag (clone M2; Sigma-Aldrich) or anti-HA (clone C19F4; Cell Signaling Technology), as described below.

For direct immunoblot analysis, spleen, thymus, bone marrow, lymph nodes, brain, lung, liver, pancreas, kidney, peripheral nerve, adrenal glands, islet, intestine, and muscle were homogenized, and the solution was filtered through a sterile 40-µm nylon cell strainer (BD Biosciences). Single-cell suspensions from tissues and isolated pan B cells were lysed in buffer (1% SDS [Thermo Fisher Scientific], 1:10,000 Benzonase [Sigma-Aldrich], and 1:100 Protease Inhibitor Cocktail [Cell Signaling Technology] and in buffer A [50 mM HEPES, 2 mM MgCl_2_, and 10 mM KCl]). Protein concentration was measured using the BCA assay (Pierce). Equal amounts (∼20 μg) of protein extracts were separated by electrophoresis on 4–12% Bis-Tris mini gels (Life Technologies) and transferred to nitrocellulose membranes (Bio-Rad). After blocking in Tris-buffered saline containing 0.05% (vol/vol) Tween-20 (TBS-T) with 5% (wt/vol) BSA at room temperature for 2 h, the membrane was incubated overnight with primary antibody (anti-Flag [clone M2; Sigma-Aldrich], anti-BiP [clone C50B12; CST], anti-IRE1α [clone 14C10; CST], anti-XBP1 [ab220783; Abcam], anti-PERK [clone C33E10; CST], anti-phospho-PERK [clone 16F8; CST], anti-phospho-eIF2α [clone D9G8; CST], anti-ATF4 [clone D4B8; CST], anti-ATF6 [clone D4Z8V; CST], anti-CHOP [clone D46F1; CST], anti-IRF4 [clone D9P5H; CST], anti-c-Myc [clone D84C12; CST], anti-Blimp-1 [clone C14A4; CST], anti-IRF8 [clone E8X4K; CST], anti-ubiquitin [clone sc-8017; Santa Cruz Biotechnology], anti-α-tubulin [clone 9F3; CST], anti-LaminA/C [clone #2032; CST], anti-PSMB5 [clone D1H6B; CST], anti-HA [clone C19F4; Cell Signaling Technology], and anti-GAPDH [clone D16H11; CST]) at 4°C in 5% (wt/vol) BSA in TBS-T with gentle rocking. The membrane was then incubated with a secondary antibody (goat anti-mouse IgG-HRP [Southern Biotech] or goat anti-rabbit IgG-HRP [Thermo Fisher Scientific]) for 1 h at room temperature. The chemiluminescence signal was developed by using a SuperSignal West Dura Extended Duration Substrate kit (Thermo Fisher Scientific) and detected by a G:Box Chemi XX6 system (Syngene).

### Hematoxylin and eosin (H&E) staining

Bones were decalcified before staining. H&E staining was performed using standard procedures by the UT Southwestern Histology Core.

### Identification of MIDN endogenous interacting candidates by liquid chromatography–tandem mass spectrometry (LC-MS/MS)

Spleens were harvested from *Midn*^*Flag/Flag*^ mice, homogenized, and the solution was filtered through a sterile 40-µm nylon cell strainer (BD Biosciences). *Midn*^*Flag/Flag*^ splenic B cells were negatively enriched using the EasySep Mouse Pan-B cell Isolation Kit (StemCell Technologies). A single cell suspension from one whole spleen or isolated pan B cells was lysed in NP-40 lysis buffer with 1:100 Protease Inhibitor Cocktail (Cell Signaling Technology). Protein concentration in the supernatant was measured using the BCA assay (Pierce).

Immunoprecipitation was performed using anti-FLAG M2 agarose beads (Sigma-Aldrich) for 4 h at 4°C, and beads were washed five times in NP-40 lysis buffer. The proteins were eluted with 100 μg/ml 3xFlag peptides at 4°C for 30 min. Proteins were precipitated in 23% trichloroacetic acid overnight at 4°C and washed with cold acetone. Proteins were solubilized in 8 M urea 100 mM triethylammonium bicarbonate pH 8.5 and reduced with 5 mM Tris (2-carboxyethyl) phosphine hydrochloride (product C4706; Sigma-Aldrich) and alkylated with 55 mM 2-chloroacetamide (product 22790; Sigma-Aldrich). Proteins were digested for 18 h at 37°C in 2 M urea 100 mM triethylammonium bicarbonate pH 8.5, with 0.5 μg trypsin (product V5111; Promega). Single-phase analysis was performed using a Q Exactive HF Orbitrap Mass Spectrometer (Thermo Fisher Scientific).

Protein and peptide identification were done with MSFragger (version 3.4) (Kong et al., 2017) (https://fragpipe.nesvilab.org/) using a mouse protein database downloaded from UniProt (http://uniprot.org) (9/1/2022, 21,992 entries), common contaminants and reversed sequences were added. The search space included all fully tryptic peptide candidates with a fixed modification of 57.021464 on C, variable modification of 15.9979 on M, and 42.0106 on the N terminus. MS1 quantification was done with total intensity and no match between runs. Protein intensity values were combined for replicates. MIDN candidate interactors identified by MS were summarized by a Venn diagram generated using http://www.biovenn.nl/index.php (Hulsen et al., 2008).

### Proteasome activity in cell lysates

Bone marrow, lung, brain, splenic B cells, liver, thymus, and pancreas were homogenized, and the solution was filtered through a sterile 40-µm nylon cell strainer (BD Biosciences). Single-cell suspensions from tissues and isolated B cells were lysed in NP-40 lysis buffer without protease inhibitors. 3 μg of total protein was added to the Proteasome Fluorescence Kit (South Bay Bio), and hydrolysis of the substrate (Suc-LLVY-AMC) was determined according to the manufacturer’s instructions.

### Generation of *Midn* knockout Sp2 cells using the CRISPR-Cas9 system

To generate *Midn*-KO Sp2 cell lines, Sp2 cells (hybridoma cell line) were transfected with a PX458 plasmid encoding a small base-pairing guide RNA targeting the genomic locus of mouse *Midn* (5′-AAC​GGA​CTG​TTC​CGG​CCT​CG-3′) and green fluorescent protein (GFP). 48 h after transfection, GFP^+^ cells were sorted by flow cytometry and single colonies were selected by a limiting dilution assay. The single colonies were screened and a 1-bp deletion was confirmed by capillary sequence (bracketed G deleted: 5′-GTC​CCT​CTC​TTT​GTA​GTC​CCA​GGC​CTC​GA[G]GCC​GGA​ACA​GTC​CGT​TAT​G-3′; chr. 10:79987479-79987527, GRCm39). The deletion is predicted to cause frameshifted protein translation after amino acid 112 and premature termination after amino acid 124 of all isoforms of MIDN.

### Standard growth curve

Live *Midn*^*−/−*^ Sp2/0 cells fused with splenic UBC-Cre-ER^T2^;*Midn*^*fl/fl*^ or *Midn*^*fl/fl*^ B cells (1 × 10^5^/well) were seeded in a 96-well plate. 4-hydroxytamoxifen (SML1666; Sigma-Aldrich) was dissolved in 95% ethanol/5% isopropanol at a concentration of 13 mM. After 24 h in culture, the cells were treated for 24 h with 4-hydroxytamoxifen at a concentration of 8.3 µM or with vehicle (95% ethanol and 5% isopropanol) diluted in the same manner as 4-hydroxytamoxifen. Every 24 h, the cells were centrifuged at 700 × *g* for 5 min and resuspended in 20 µl PBS. Suspended cells (10 μl) and an equal volume of Trypan Blue (10 μl) were mixed to count the total number of live cells using a TC20 Automated Cell Counter (Bio-Rad). A standard growth curve was generated by graphing cell counts every 24 h.

### Plasmids

Mouse *Midn* (NM_021565.2) was tagged N-terminally with an HA epitope in a pCMV6 vector. Full-length mouse PSMC2 (NM_011188.4), PSMC4 (NM_011874.2), PSMC5 (NM_008950.1), or PSMD11 (NM_178616.3) with FLAG epitope were cloned into pCDNA6 vector.

The cDNA sequence of human wild-type *MIDN* (1–468 aa) was synthesized (IDT) and used as a template to clone hMIDN^WT^, point mutants (hMIDN-*Midnight*, hMIDN-*full_moon*), and truncated versions of hMIDN (hMIDN-ΔC-term, hMIDN-UBL, hMIDN-ΔUBL) into a custom pET-derived vector for bacterial overexpression. Except for hMIDN-UBL, the inserts also contained an N-terminal 14× histidine followed by a bdNEDD8 protease cleavage site. hMIDN-UBL was cloned into the pET-derived vector with an N-terminal 6× histidine fused to an MBP tag followed by the TEV protease cleavage site. The *Midnight* mutation (S281P) and *full_moon* mutation (R12L) of MIDN were generated by site-directed mutagenesis using the QuikChange II site-directed mutagenesis kit (Agilent Technologies). All plasmids were sequenced to confirm the absence of undesirable mutations.

### Expression and purification of recombinant MIDN and variants

Customized pET-derived vectors encoding human wild-type MIDN, MIDN-Midnight, MIDN-full_moon, MIDN-ΔC-term, MIDN-UBL, or MIDN-ΔUBL (Fig. 2 F) were transformed into *Escherichia coli* BL21 Rosette (DE3) cells. Cells were grown at 37°C to an OD_600_ of 0.8 and induced with a final concentration of 0.25 mM IPTG (isopropyl-β-D-thiogalactoside) at 18°C overnight. After harvesting cells by centrifugation for 20 min at 4,000 × *g*, cell pellets were resuspended in B1 buffer (50 mM Tris pH 7.5, 800 mM NaCl, 40 mM imidazole, 10% glycerol) with complete protease inhibitors (Thermo Fisher Scientific Pierce Protease Inhibitor Tablets) and lysed via sonication. The lysate was cleared by centrifuging for 40 min at 18,000 × *g* and the cleared lysate was loaded onto a 5 ml Hi-Trap Ni-NTA column (Cytiva), washed with five-column volumes of buffer B1 and subsequently eluted with two-column volumes of buffer B2 (50 mM Tris pH 7.5, 100 mM NaCl, 400 mM imidazole, 10% glycerol) and loaded directly onto a 5 ml Hi-Trap SP column (Cytiva). Protein was eluted using a linear gradient of buffer A1 (50 mM Tris pH 7.5, 50 mM NaCl, 40 mM imidazole, 10% Glycerol) and buffer B1 spanning 10 column volumes. The main peak fractions eluted from ion exchange were pooled, affinity/solubility tags were cleaved overnight with bdNEDD8p protease, and the proteins were each purified by size exclusion chromatography on an S200 16/60 equilibrated in gel filtration buffer (30 mM Tris pH 7.5, 150 mM NaCl, 1 mM Tris(2-carboxyethal)phosphine). Purity was assessed by Coomassie-stained SDS-PAGE, and aliquots were concentrated using Amicon Ultra spin columns (Merck Millipore) with 30 kDa cutoff and snap-frozen in liquid nitrogen for later use.

### Proteasome stimulating activity assay

The proteasome stimulating activity of MIDN toward human proteasomes (26 and 20S) was measured using fluorogenic substrate Suc-LLVY-AMC (South Bay Bio). For 26S proteasome stimulating activity assay, human 26S proteasomes (Cat. #A1100; UBPBio, purified from HEK293 cells with no detectable *Midn* transcript expression) (1 nM) were incubated with hMIDN-WT, hMIDN-*Midnight*, hMIDN-*full_moon*, hMIDN-ΔC-term, hMIDN-UBL, or hMIDN-ΔUBL in buffer (50 mM Tris-HCl pH 7.5, 100 mM KCl, 0.5 mM MgCl_2_, 1 mM TECP, 0.2 mM ATP, and 25 ng/μl BSA) for 5 min at room temperature. 10 μM Suc-LLVY-AMC (South Bay Bio) was added to the reaction mixture and the fluorescent reaction product (AMC) was detected with a BioTek Multimode Plate Reader at 345 nm/445 nm excitation/emission over a 1.5 h time course. The 1.5 h time point was used for the calculation of proteasome activity. For the 20S proteasome stimulating activity assay, purified MIDN or its variants were added to the reaction mixture in the 20S Proteasome Kit (South Bay Bio), and hydrolysis of the substrate (Suc-LLVY-AMC) was determined according to the manufacturer’s instructions.

### Blood glucose assay

Blood glucose was measured with the AlphaTRAK glucometer and test strips.

### In vitro B cell class switching and proliferation analysis

Splenic naïve B lymphocytes were isolated from Mb1-Cre;*Midn*^*fl/fl*^ or wild-type littermates using anti-CD43 MicroBeads (Miltenyi Biotec). Cells were stained with 5 µM CellTrace CFSE (Life Technologies) and stimulated to undergo class switching to IgG1 with 25 μg/ml LPS (Sigma-Aldrich) and 10 ng/ml of mouse recombinant IL-4 (BioLegend) for 72 h at 37°C. In vitro class switching and proliferation rate were analyzed by flow cytometry.

### Hematological analysis

Hematological analysis of the peripheral blood was performed using a HemaVet 950FS Hematology Analyzer (Drew Scientific).

### Tamoxifen administration for Cre induction from UBC-Cre-ER^T2^ transgene

Tamoxifen (#T5648; Sigma-Aldrich) was dissolved in corn oil at a concentration of 20 mg/ml by shaking overnight at 37°C in the dark. Tamoxifen/corn oil solution was stored in the dark at 4°C for the duration of injections. 100 µl of tamoxifen/corn oil solution was injected intraperitoneally in adult mice once every 24 h for a total of five consecutive days.

### Blood smears

Approximately 3–4 µl of freshly drawn blood was placed on a clean, labeled slide and spread to a thin film using a second clean slide. After air drying, blood cells were fixed by submerging the slide in methanol for 30 s. The slides were then placed in a Wright-Giemsa Stain for 2.5 min. The slides were then moved to a Stain/Buffer mixture for an additional 2 min. Lastly, the slides were rinsed in Milli-Q ultrapure water for 2 min and allowed to fully air dry. Cover slips were placed with mounting medium, and the slides were left to dry overnight in the dark.

### Statistical analysis

The statistical significance of differences between groups was analyzed using GraphPad Prism by performing the indicated statistical tests. Differences in the raw values among groups were considered statistically significant when P < 0.05. P values are denoted by * P < 0.05; ** P < 0.01; *** P < 0.001; NS, not significant with P > 0.05.

### Online supplemental material

Fig. S1 shows the offspring from *Midn*^*+/Mid*^ or *Midn*^*+/KD*^ crosses, the design of *Midn*^*fl*^ and *Midn*^*KD*^ alleles, and complete blood counts, body weight, and bone phenotypes in *Midn*^*KD/KD*^ mice. Fig. S2 shows increased proliferation and class switching in vitro by MIDN-deficient B cells compared with WT B cells, and serum immunoglobulin concentration and relative numbers of splenic B cell populations in *Midn*^*KD/KD*^ mice. Fig. S3 shows endogenous MIDN tissue expression and subcellular compartmentalization, and blood glucose concentration in *Midn*^*KD/KD*^ mice. Fig. S4 shows an alignment of human and mouse MIDN amino acid sequences. Fig. S5 shows plasma cell production after immunization with the TD antigen β-galactosidase in Mb1-Cre;*Midn*^*fl/fl*^ mice. Table S1 shows primer sequences. Data S1 shows mass spectrometry analysis of proteins immunoprecipitated with endogenous FLAG-tagged MIDN in B cell lysate. Data S2 shows mass spectrometry analysis of proteins immunoprecipitated with endogenous FLAG-tagged MIDN in spleen lysate and a list of MIDN interactors summarized in Fig. 5 C (Venn diagram).

## Supplementary Material

Table S1 shows primer sequences.

Data S1 shows mass spectrometry analysis of proteins immunoprecipitated with endogenous FLAG-tagged MIDN in B cell lysate.

Data S2 shows mass spectrometry analysis of proteins immunoprecipitated with endogenous FLAG-tagged MIDN in spleen lysate and a list of MIDN interactors summarized in Fig. 5 C (Venn diagram).

SourceData F5 contains original blots for Fig. 5.

SourceData F6 contains original blots for Fig. 6.

SourceData F7 contains original blots for Fig. 7.

SourceData F8 contains original blots for Fig. 8.

SourceData FS3 contains original blots for Fig. S3.

## Data Availability

The raw mass spectrometry data underlying Fig. 5, A–C are openly available from the ProteomeXchange database with accession no. PXD046953.
