# Supplementary material for: Viable mutations of mouse midnolin suppress B cell malignancies
Source: J Exp Med. 2024 Apr 16;221(6):e20232132. doi: 10.1084/jem.20232132 (PMC11022886; doi:10.1084/jem.20232132)
Supplement: Table S1 — shows primer sequences. [file JEM_20232132_TableS1.docx]

**Table S1. Primer sequences**

| Gene | Forward primer (5’ to 3’) | Reverse primer (5’ to 3’) |
| --- | --- | --- |
| Midn (PCR) | TTTTGCAGATGGGTTAGTGCTTAGTG | TCCAAGGCTTGCATAACGGACT |
| Midn  (Sequencing) | AGCTGAGGGGTGGAAAACGCTC |  |
| MIDN-3xFlag-TG | GAGACCGTCTTCGACAGACAG | CTTGTCATCGTCATCCTTGTAATCGATATCATGATC |
| MIDN-3xFlag-CS | GAGACCGTCTTCGACAGACAG | GGGAAGGGCCCTGTTGCAGAG |
| Midn-flox-up-TG | CCTCTTCCTTCCTACCCCCAG | TACGAAGTTATTCACGCAACGAA |
| Midn-flox-up-WT | CCTCTTCCTTCCTACCCCCAG | TTGCGTGCTAGGGGCAGT |
| Midn-flox-down-TG | AAGTTATCGCTTCATGTGACTCAAGC | CTTGGGAGGTATGGGCATAGG |
| Midn-flox-down-WT | CAAGCCTCCTTGGGGTTTT | CTTGGGAGGTATGGGCATAGG |
| Midn-2xHA | GAGACCGTCTTCGACAGACAG | GGGAAGGGCCCTGTTGCAGAG |
