# Supplementary figures and images for: Viable mutations of mouse midnolin suppress B cell malignancies
Source: J Exp Med. 2024 Apr 16;221(6):e20232132. doi: 10.1084/jem.20232132 (PMC11022886; doi:10.1084/jem.20232132)

IP:Flag  
IB:HA

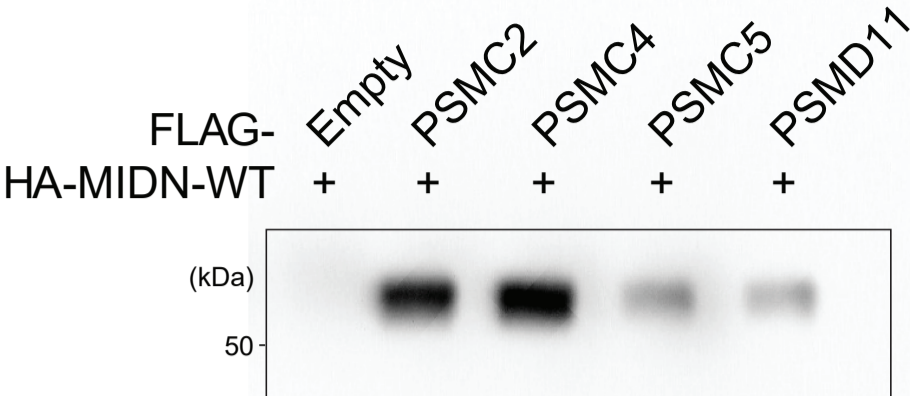

IP:Flag  
IB:Flag

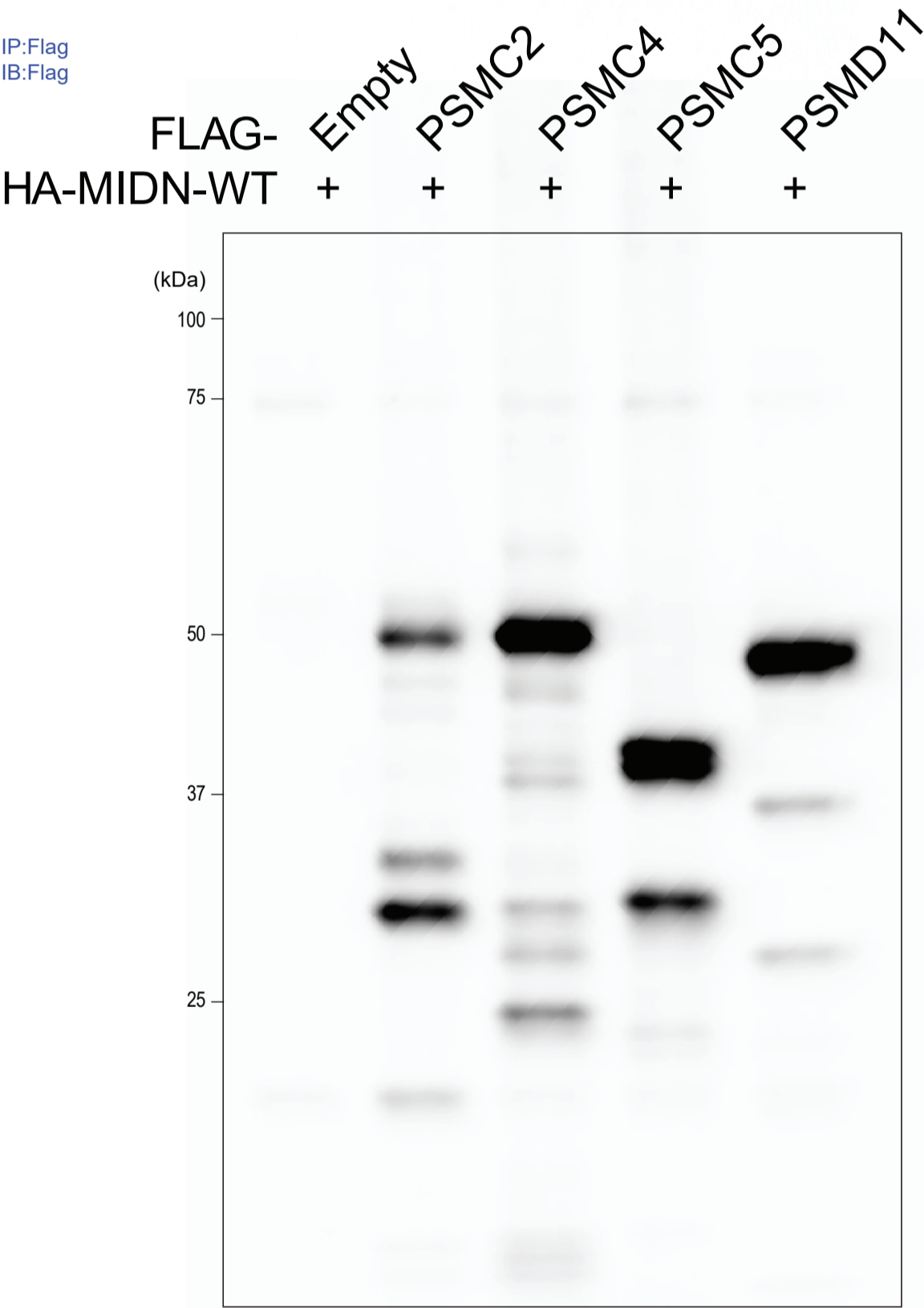

Input  
IB:HA

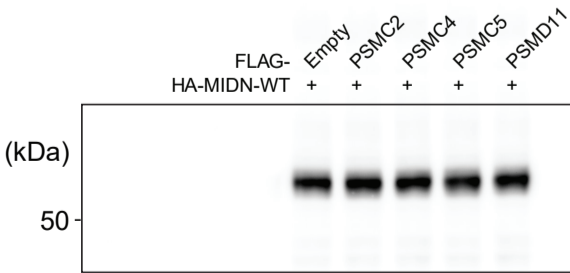

Supplement: SourceData F5 — contains original blots for Fig. 5. [file JEM_20232132_SourceDataF5.pdf]

Midn<sup>fl/fl</sup>

Ubc-Cre;Midn<sup>fl/fl</sup>

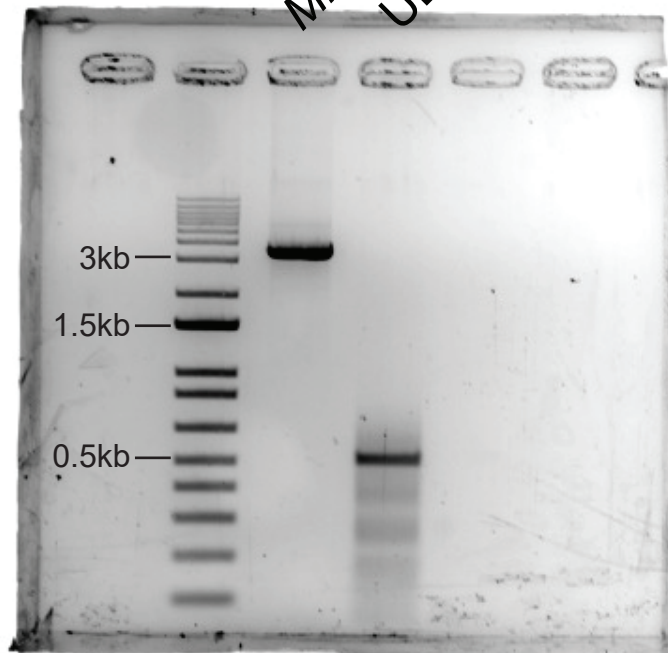

Supplement: SourceData F7 — contains original blots for Fig. 7. [file JEM_20232132_SourceDataF7.pdf]

IRF4

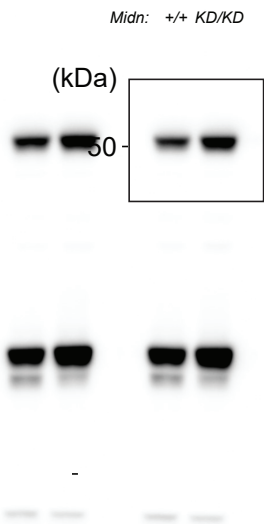

c-Myc

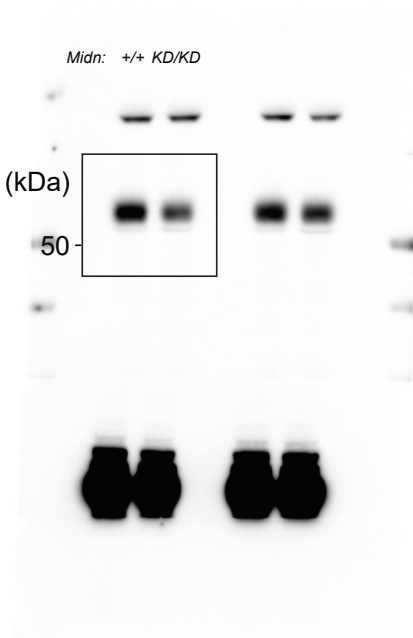

BLIMP1

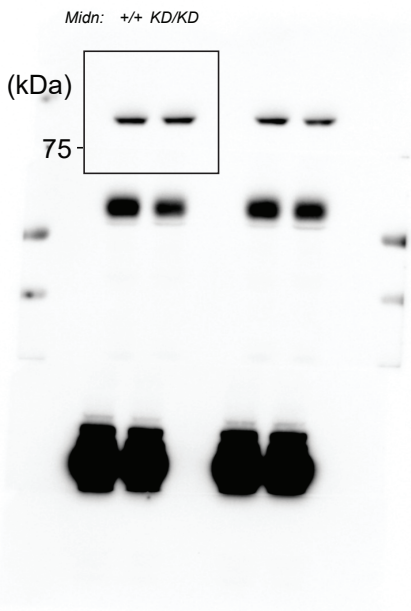

IRF8

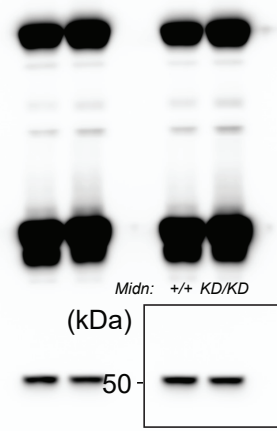

GAPDH

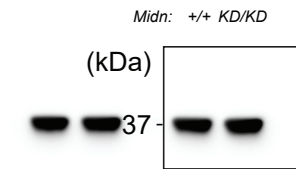

Supplement: SourceData F8 — contains original blots for Fig. 8. [file JEM_20232132_SourceDataF8.pdf]
