# Supplementary material for: Viable mutations of mouse midnolin suppress B cell malignancies
Source: J Exp Med. 2024 Apr 16;221(6):e20232132. doi: 10.1084/jem.20232132 (PMC11022886; doi:10.1084/jem.20232132)

MIDN-HA (up left)

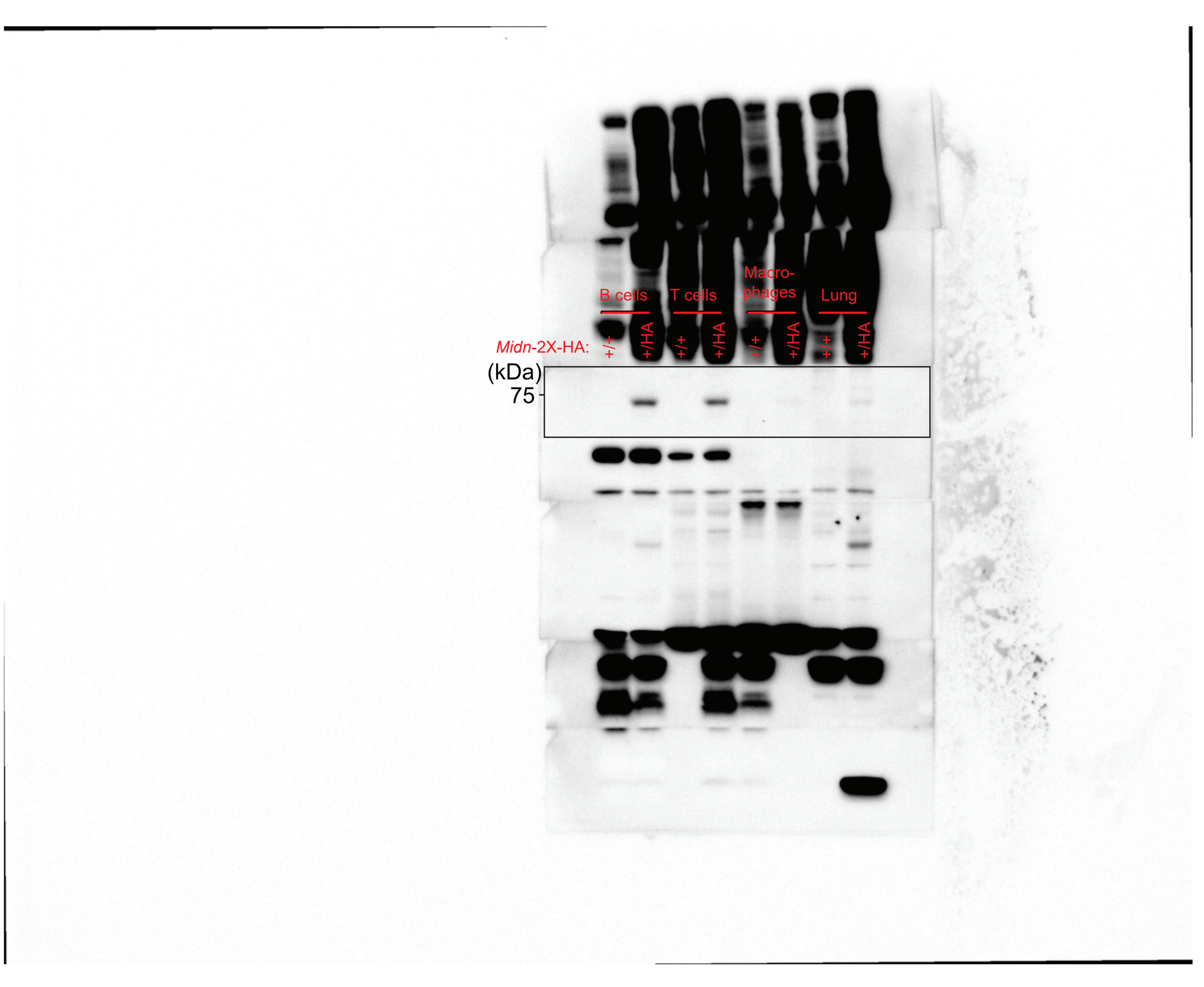

GAPDH (up left)

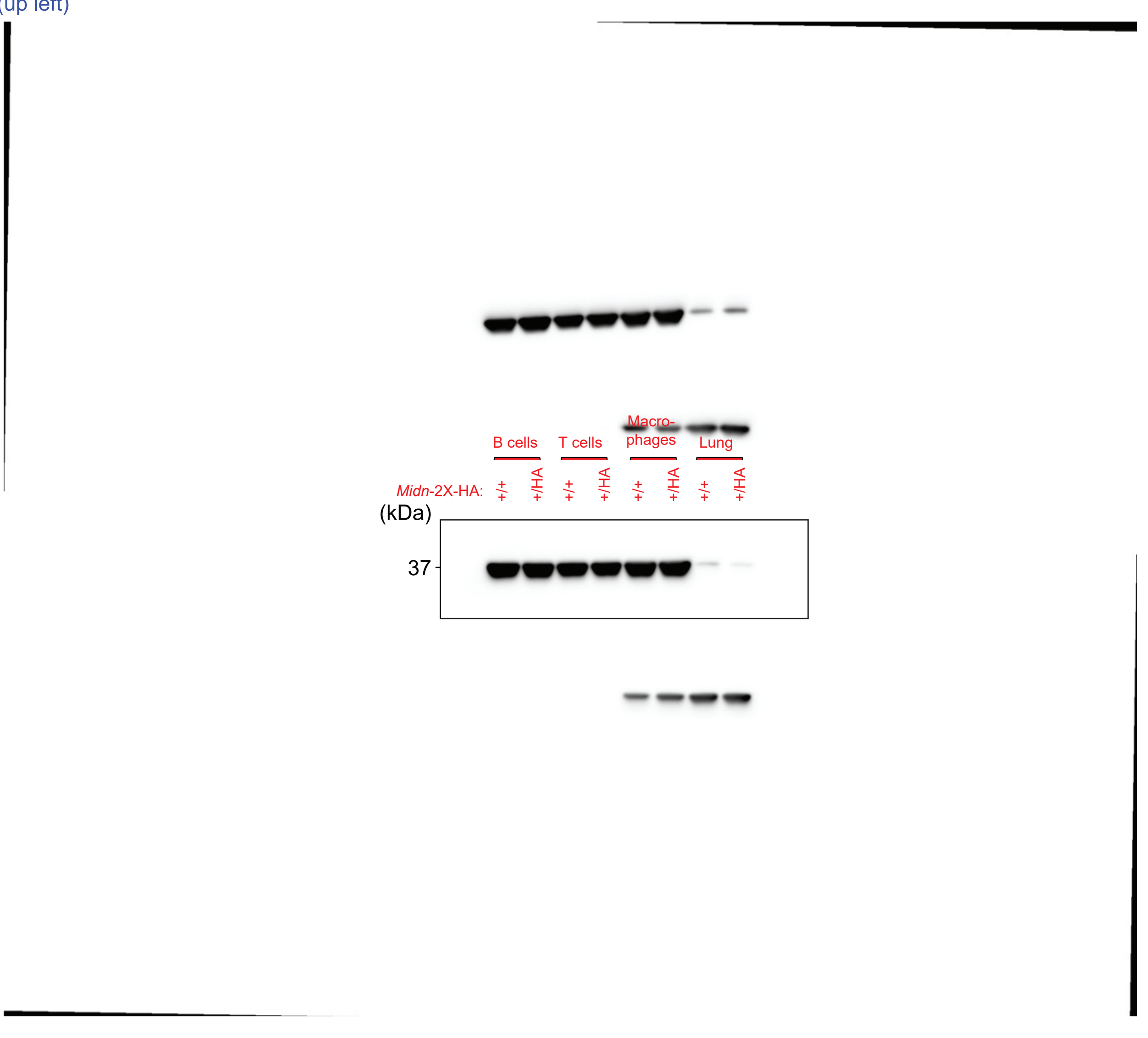

MIDN-HA (up right)

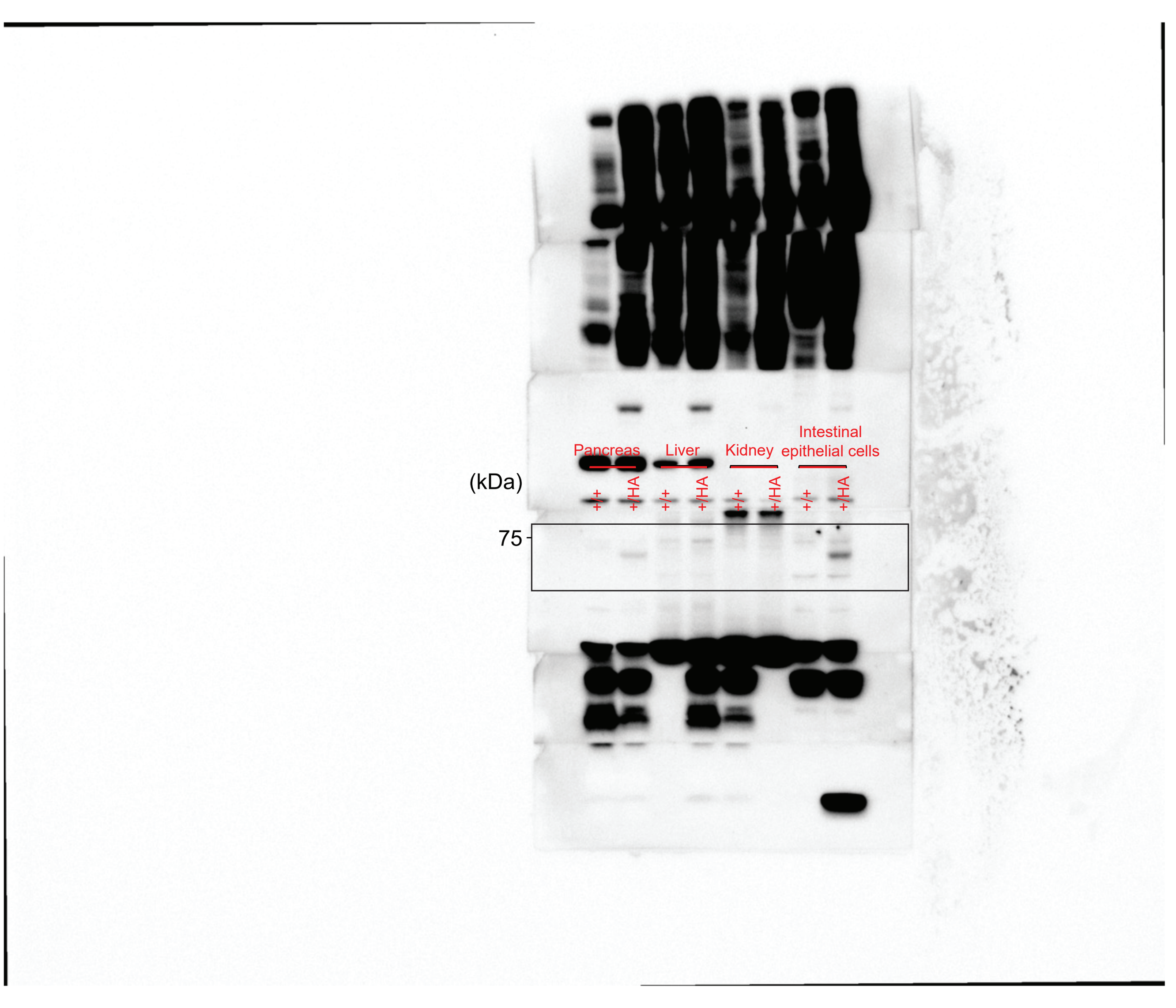

GAPDH (up right)

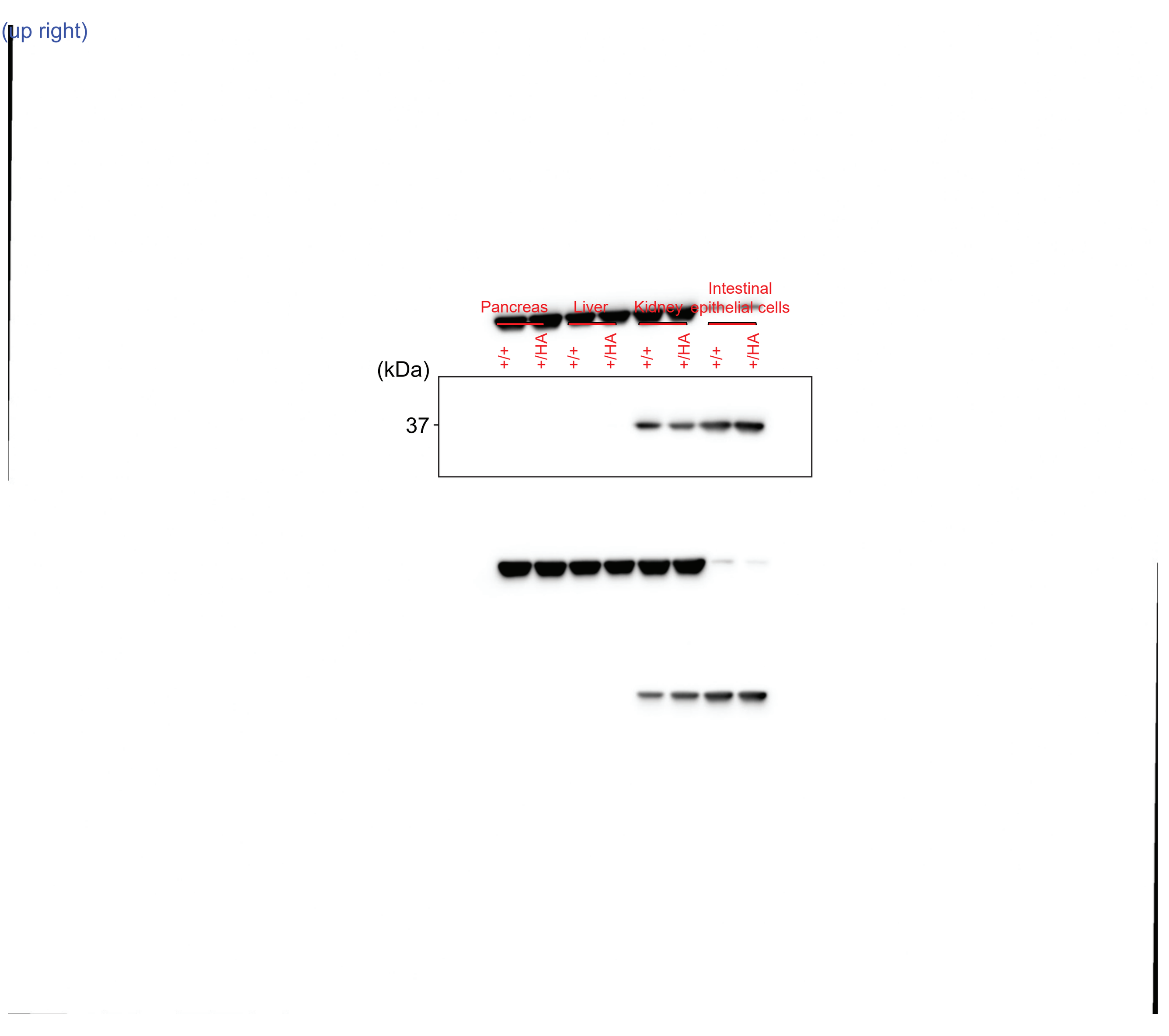

MIDN-HA (down left)

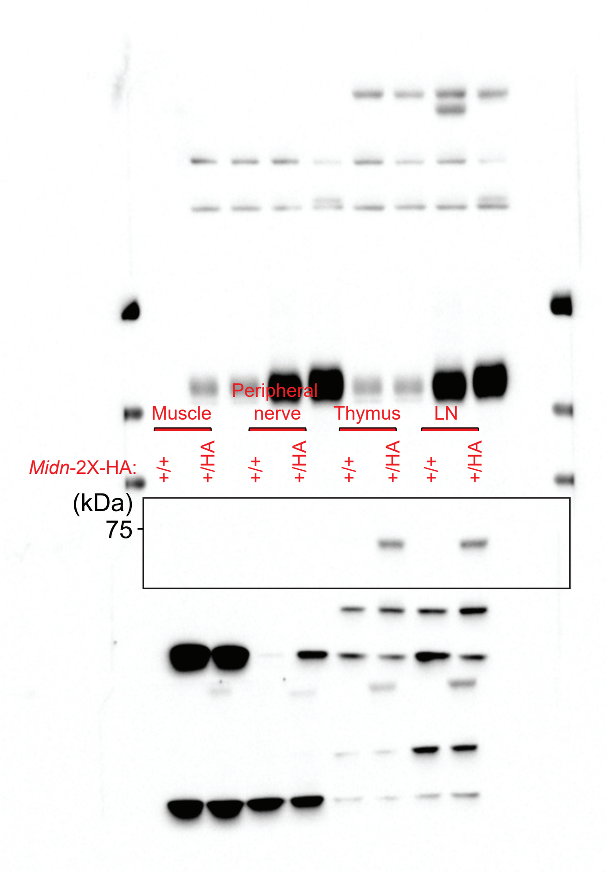

PSMB5 (down left)

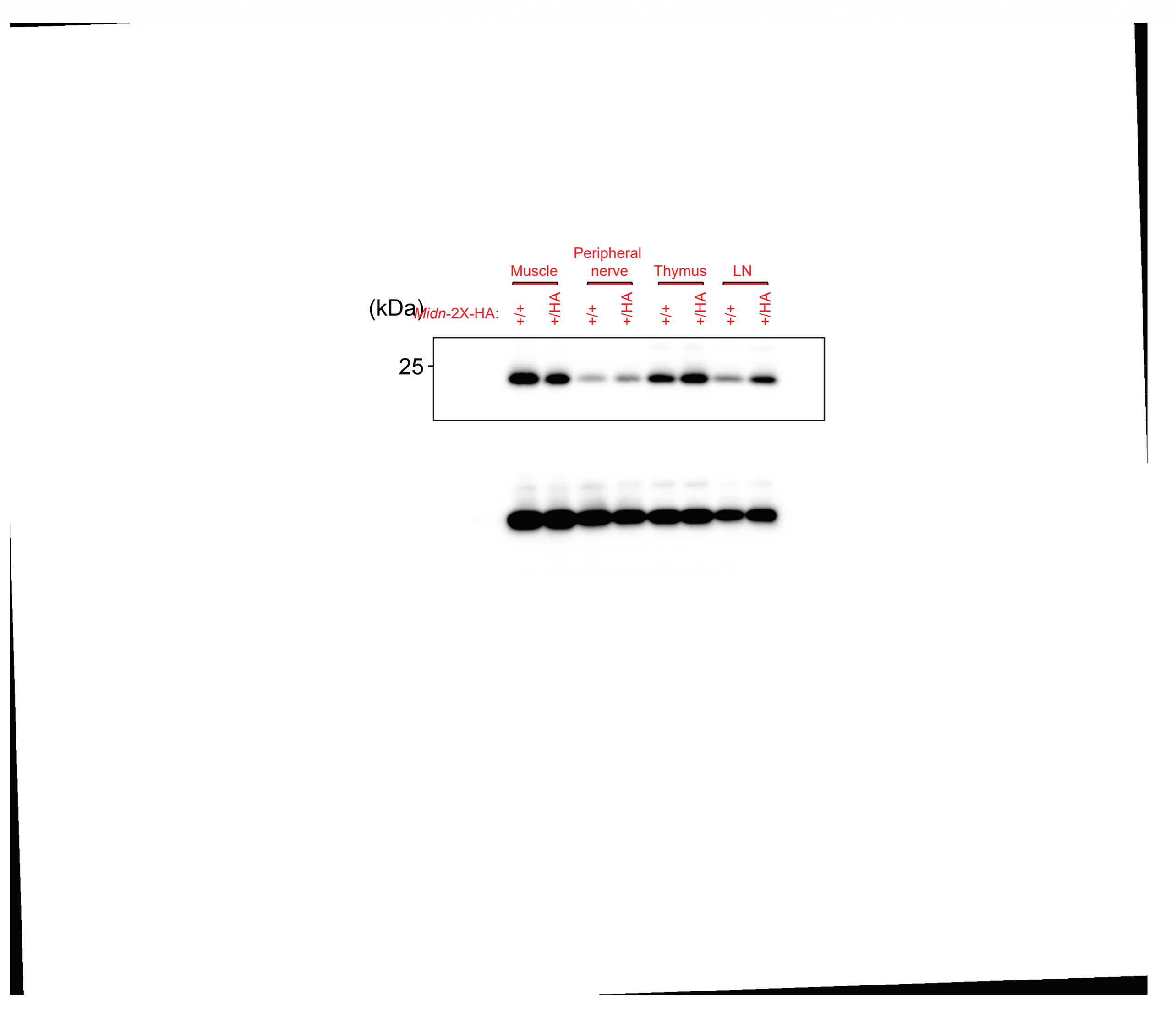

MIDN-HA (down right)

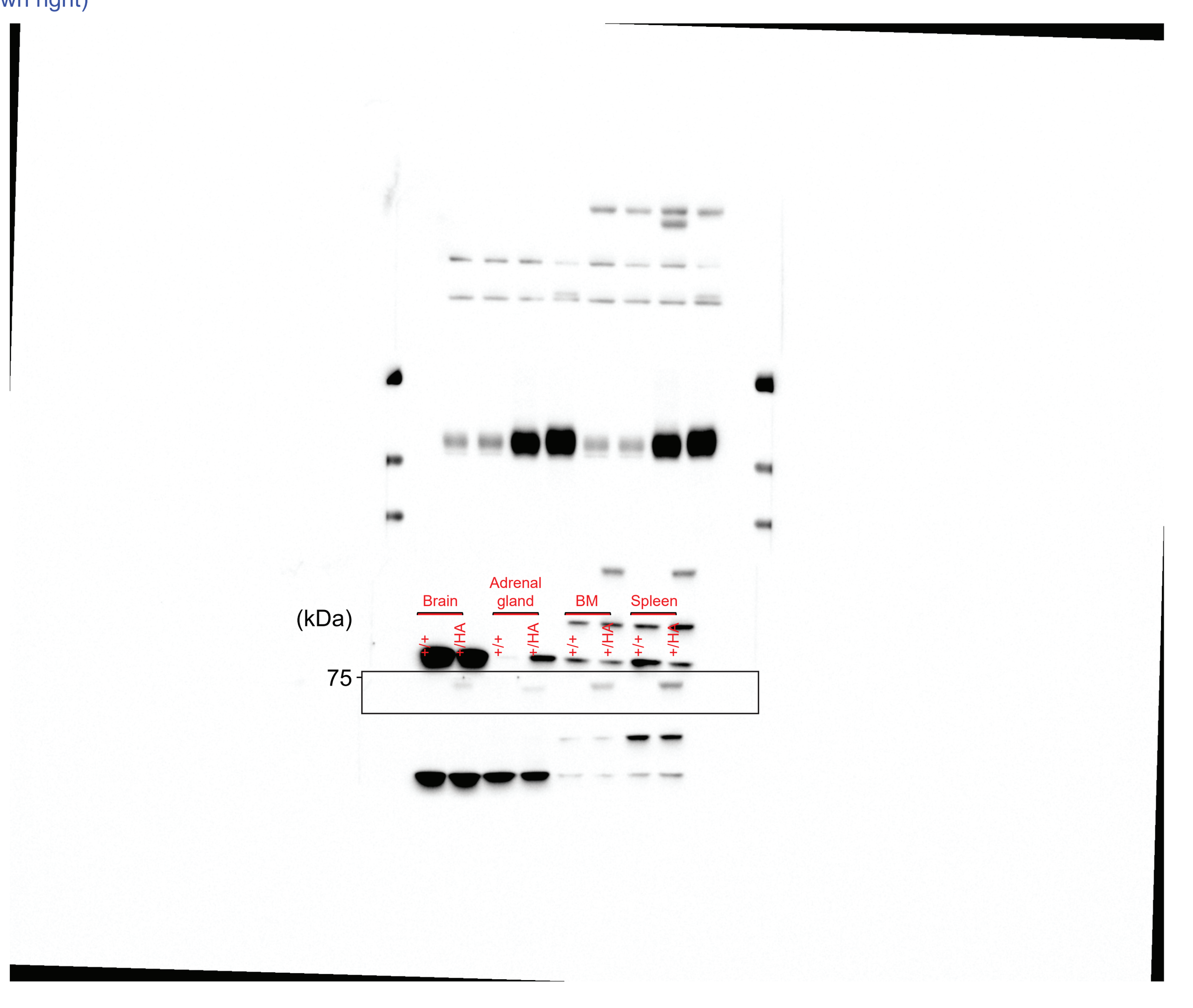

PSMB5 (down right)

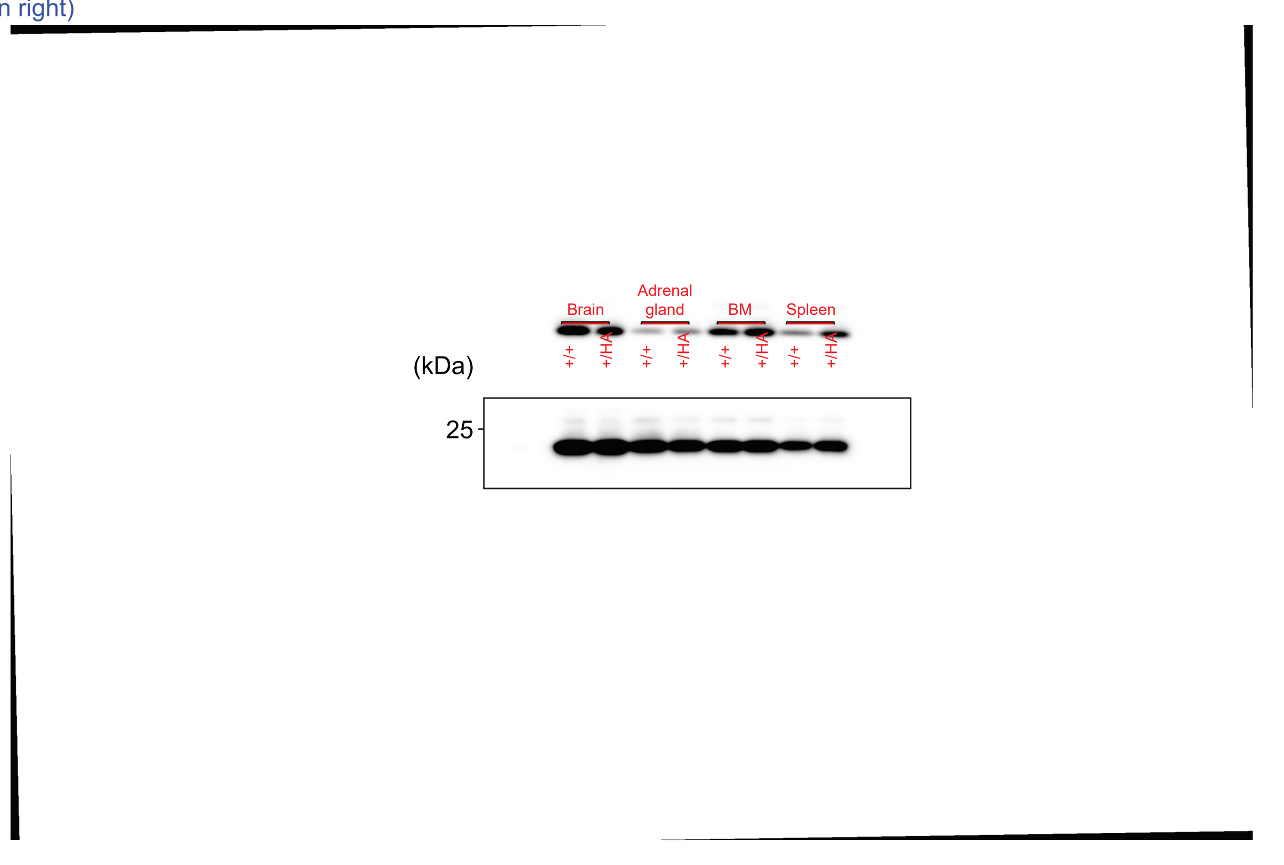

MIDN-Flag

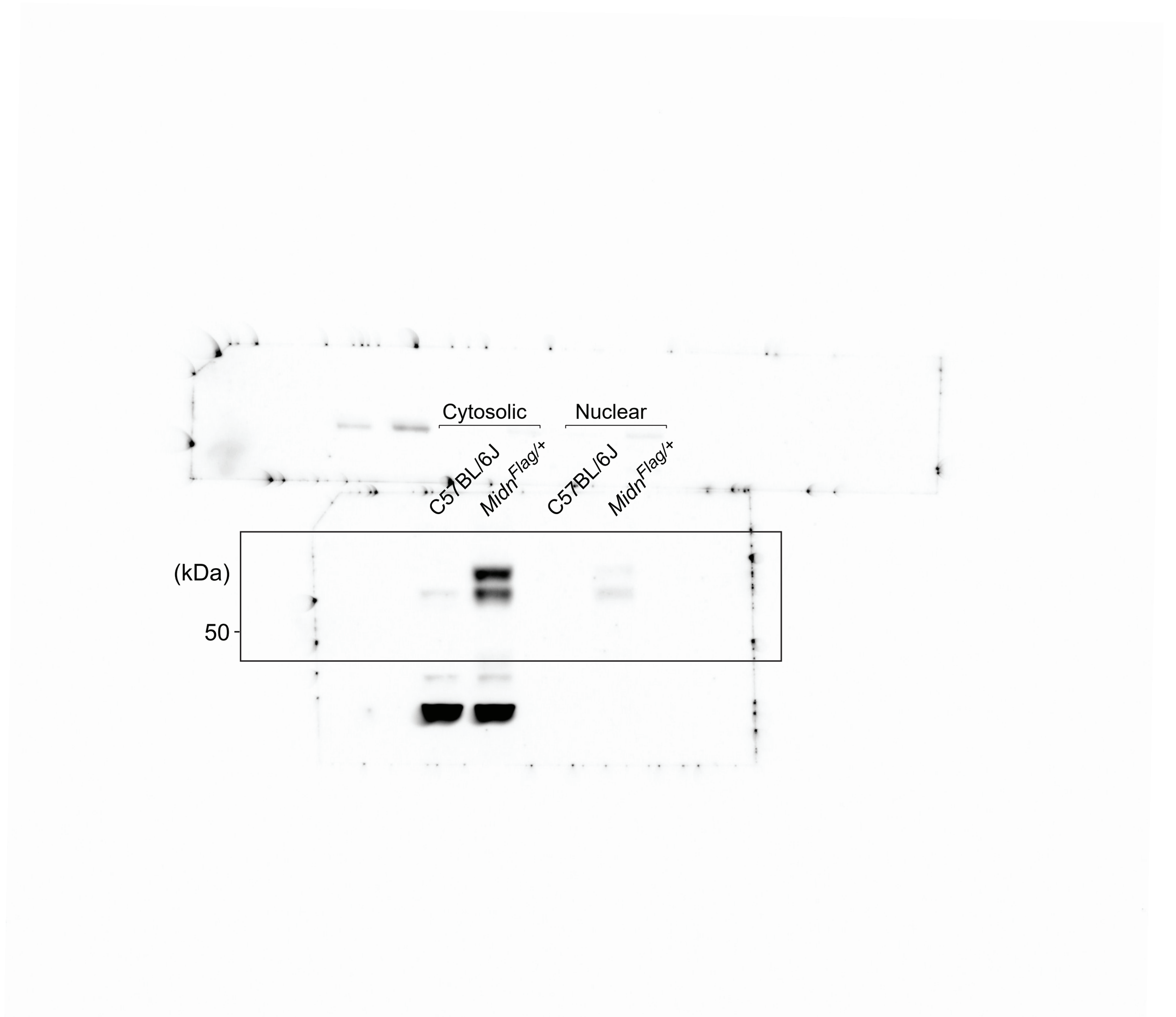

α-Tubulin

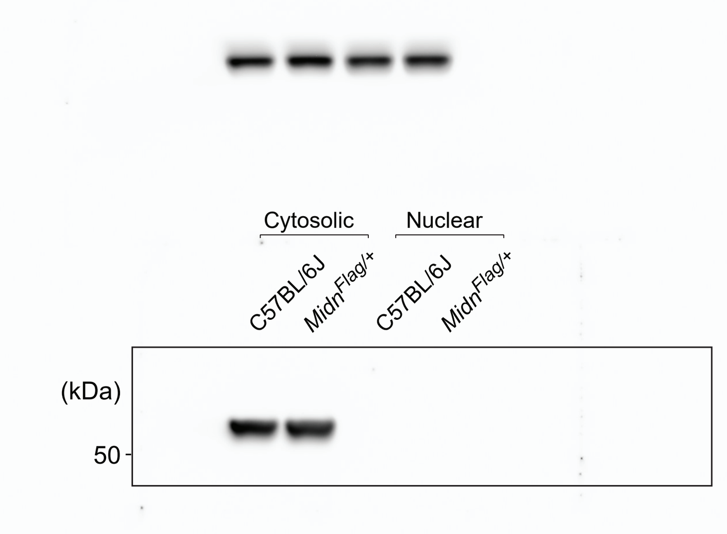

Lamin A/C

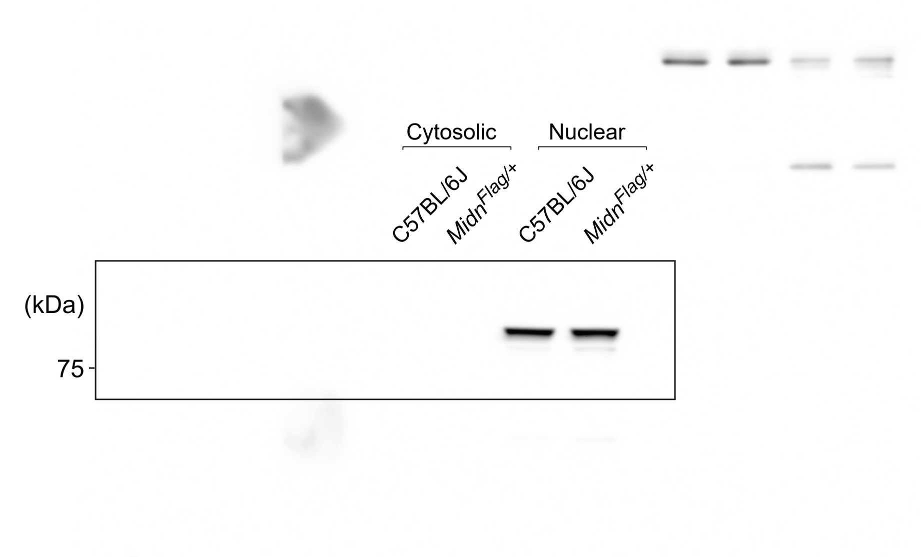

Supplement: SourceData FS3 — contains original blots for Fig. S3. [file JEM_20232132_SourceDataFS3.pdf]
