# Supplementary material for: Viable mutations of mouse midnolin suppress B cell malignancies
Source: J Exp Med. 2024 Apr 16;221(6):e20232132. doi: 10.1084/jem.20232132 (PMC11022886; doi:10.1084/jem.20232132)

Bip

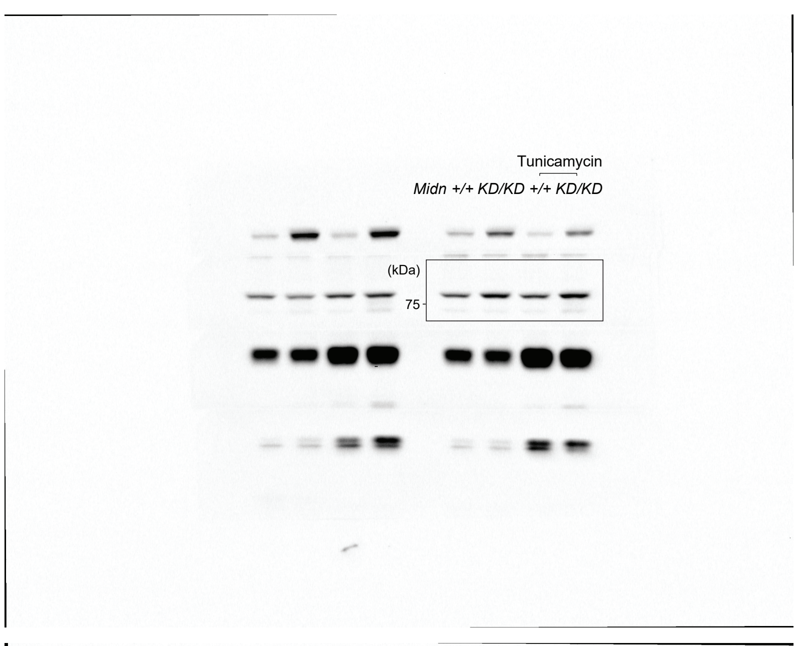

IRE1α

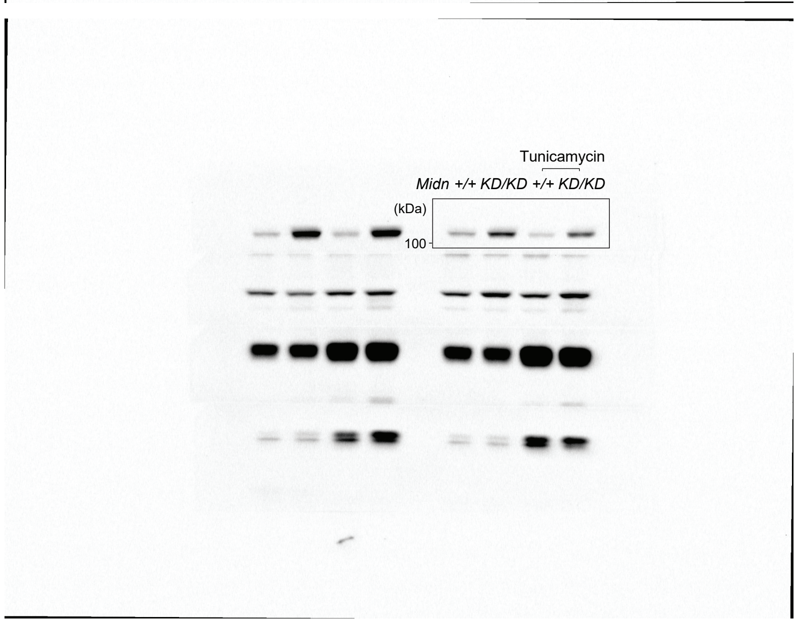

XBP1

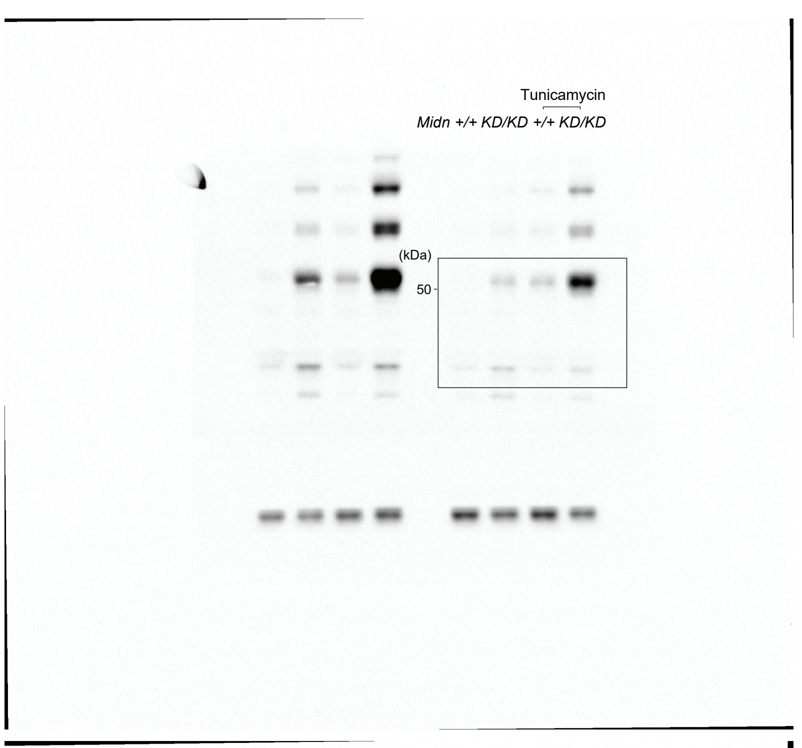

p-PERK

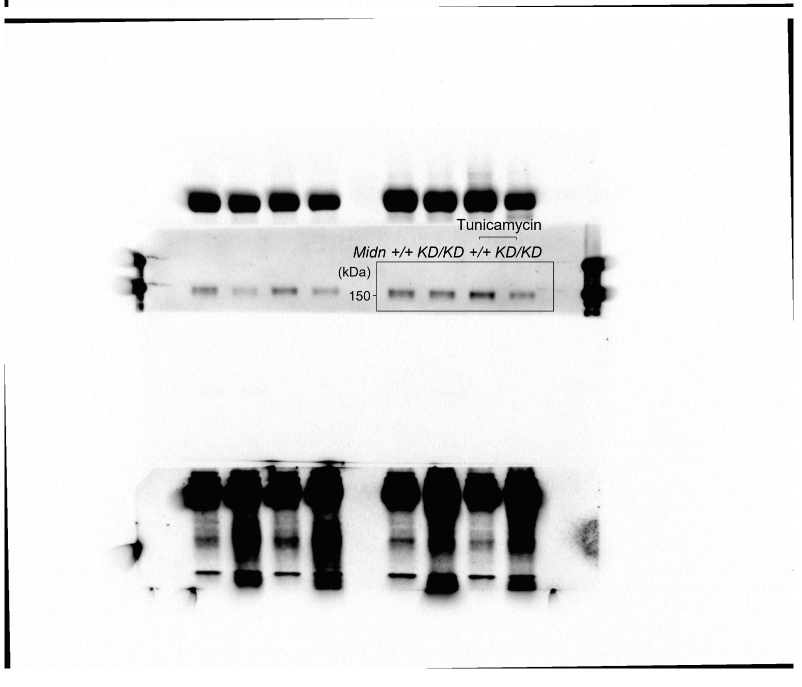

PERK

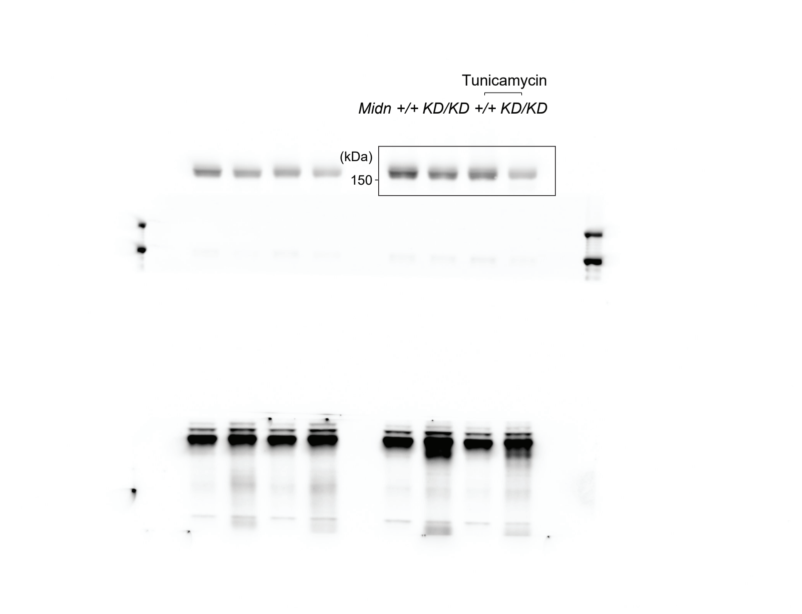

p-eIF2α

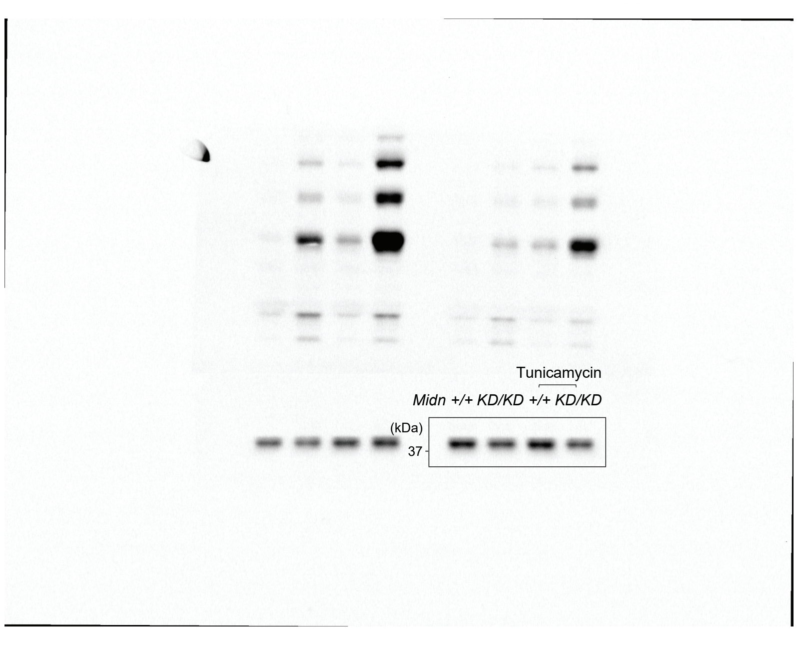

ATF4

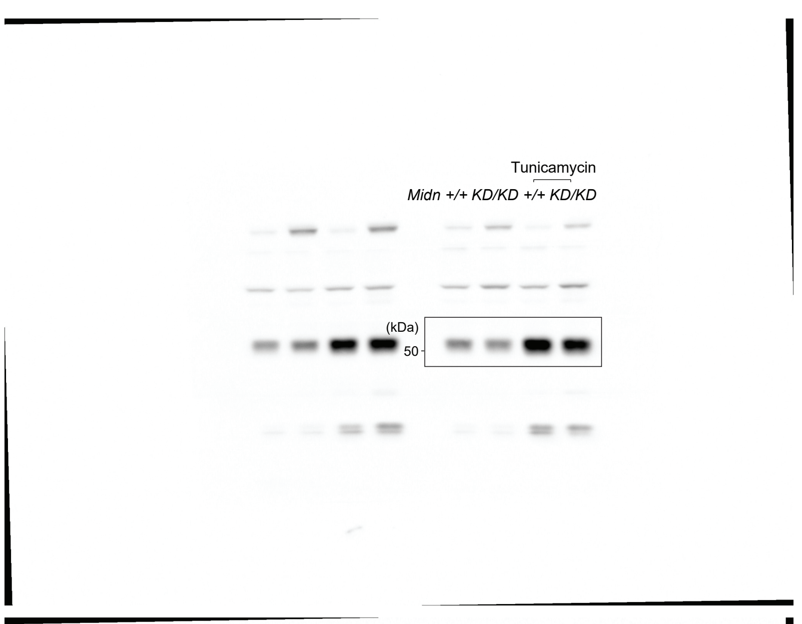

CHOP

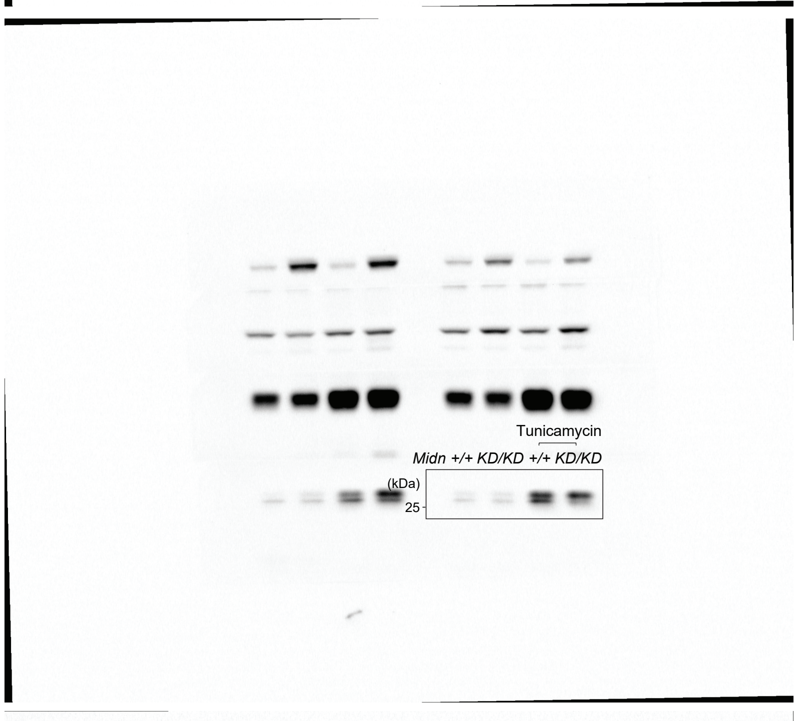

ATF6

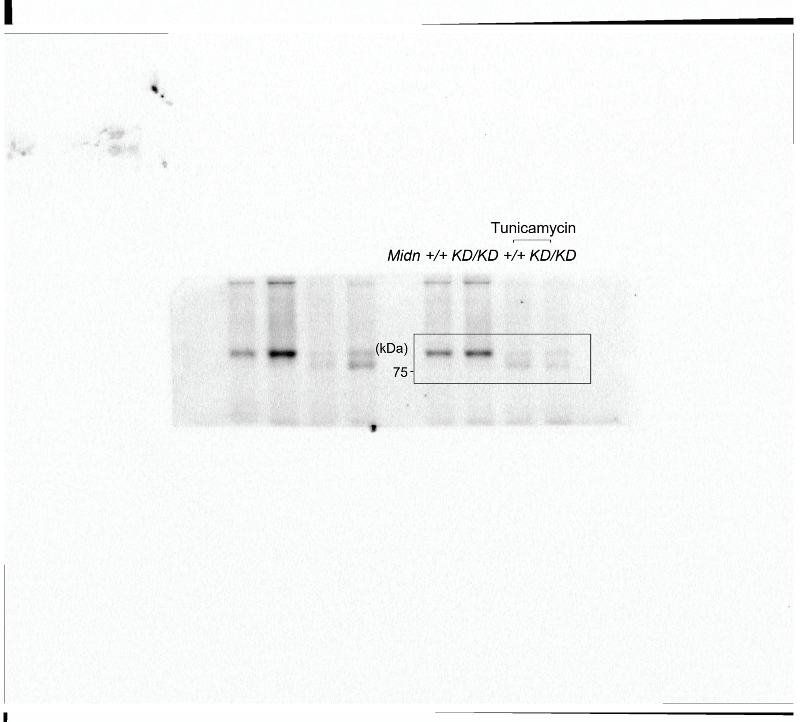

GAPDH

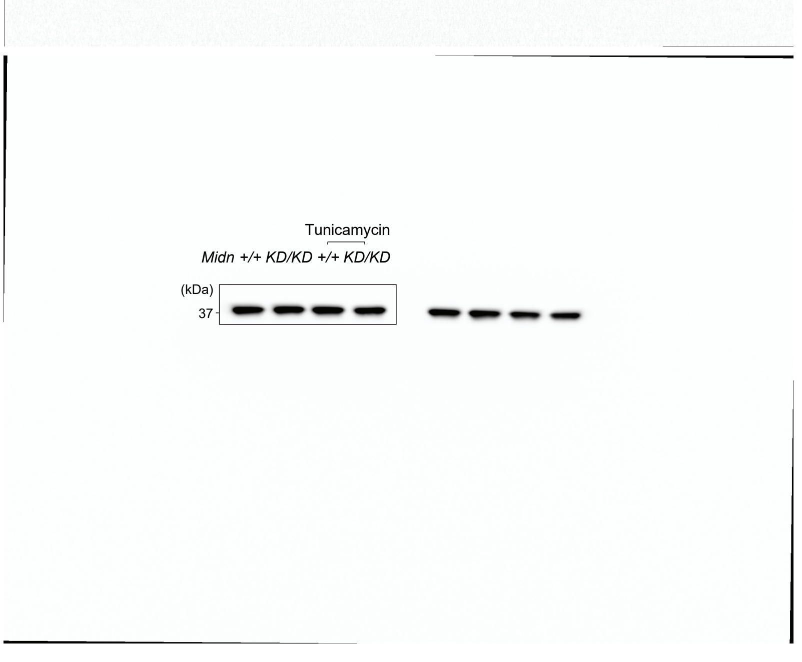

MIDN-HA

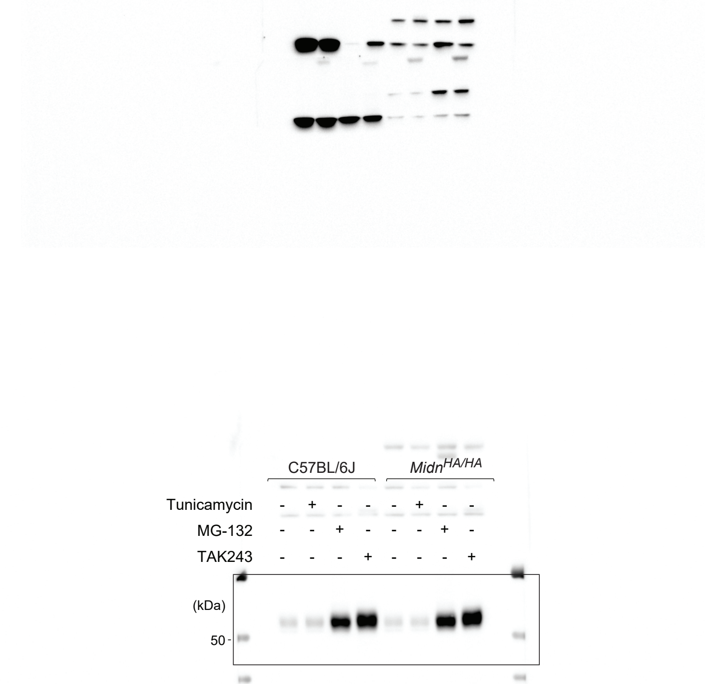

c-Myc

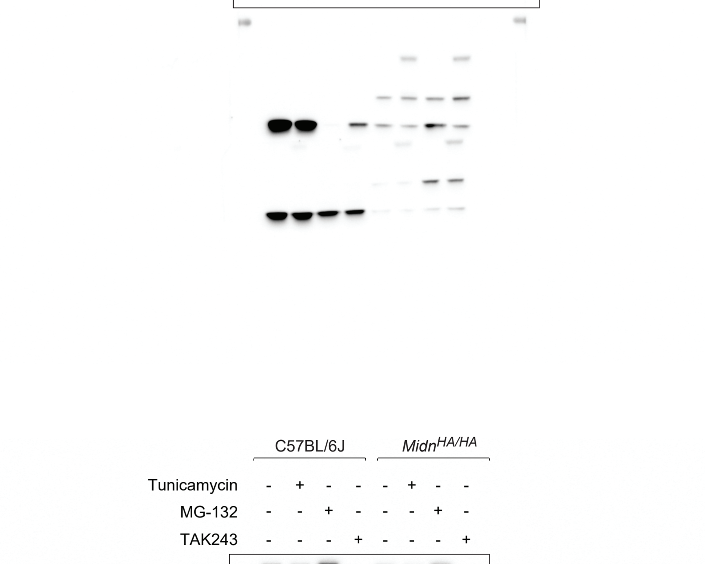

UB

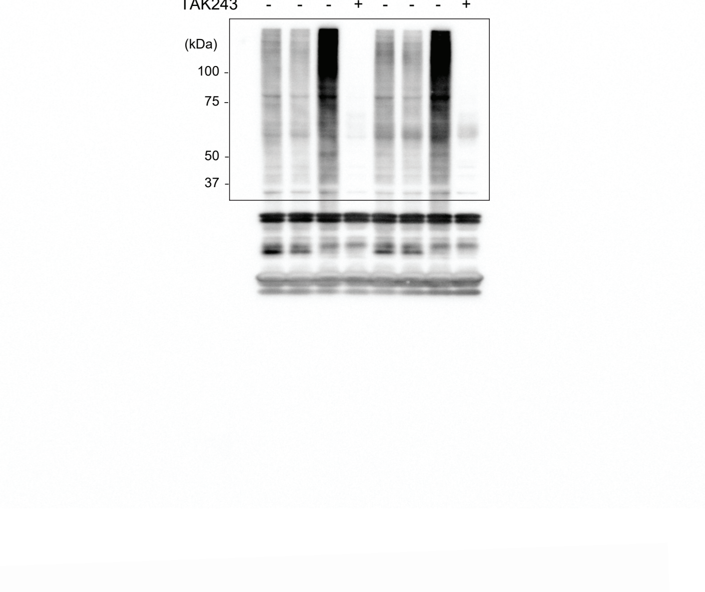

GAPDH

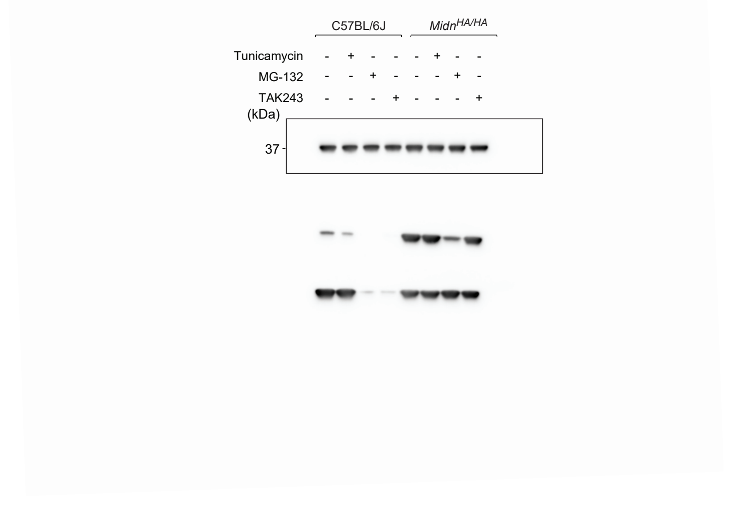

Supplement: SourceData F6 — contains original blots for Fig. 6. [file JEM_20232132_SourceDataF6.pdf]
